# Phages against Noncapsulated Klebsiella pneumoniae: Broader Host range, Slower Resistance

**DOI:** 10.1128/spectrum.04812-22

**Published:** 2023-06-20

**Authors:** Marta Lourenço, Lisa Osbelt, Virginie Passet, François Gravey, Daniela Megrian, Till Strowig, Carla Rodrigues, Sylvain Brisse

**Affiliations:** a Institut Pasteur, Université Paris Cité, Biodiversity and Epidemiology of Bacterial Pathogens, Paris, France; b Department of Microbial Immune Regulation, Helmholtz Center for Infection Research, Braunschweig, Germany; c German Center for Infection Research (DZIF), partner site Hannover-Braunschweig, Braunschweig, Germany; d Dynamycure Inserm UM1311 Normandie Univ, UNICAEN, UNIROUEN, Caen, France; e Unité de Microbiologie Structurale, Institut Pasteur, CNRS UMR 3528, Université Paris Cité, Paris, France; University of Exeter

**Keywords:** *Klebsiella pneumoniae*, bacteriophages, phage-bacteria interactions, phage therapy, noncapsulated mutants, phage resistance, antimicrobial resistance, genomics, host range, *in vivo*, bacteriophage therapy, bacteriophage-bacteria interactions

## Abstract

Klebsiella pneumoniae (Kp), a human gut colonizer and opportunistic pathogen, is a major contributor to the global burden of antimicrobial resistance. Virulent bacteriophages represent promising agents for decolonization and therapy. However, the majority of anti-Kp phages that have been isolated thus far are highly specific to unique capsular types (anti-K phages), which is a major limitation to phage therapy prospects due to the highly polymorphic capsule of Kp. Here, we report on an original anti-Kp phage isolation strategy, using capsule-deficient Kp mutants as hosts (anti-K^d^ phages). We show that anti-K^d^ phages have a broad host range, as the majority are able to infect noncapsulated mutants of multiple genetic sublineages and O-types. Additionally, anti-K^d^ phages induce a lower rate of resistance emergence *in vitro* and provide increased killing efficiency when in combination with anti-K phages. *In vivo*, anti-K^d^ phages are able to replicate in mouse guts colonized with a capsulated Kp strain, suggesting the presence of noncapsulated Kp subpopulations. The original strategy proposed here represents a promising avenue that circumvents the Kp capsule host restriction barrier, offering promise for therapeutic development.

**IMPORTANCE** Klebsiella pneumoniae (Kp) is an ecologically generalist bacterium as well as an opportunistic pathogen that is responsible for hospital-acquired infections and a major contributor to the global burden of antimicrobial resistance. In the last decades, limited advances have been made in the use of virulent phages as alternatives or complements to antibiotics that are used to treat Kp infections. This work demonstrates the potential value of an anti-Klebsiella phage isolation strategy that addresses the issue of the narrow host range of anti-K phages. Anti-K^d^ phages may be active in infection sites in which capsule expression is intermittent or repressed or in combination with anti-K phages, which often induce the loss of capsule in escape mutants.

## INTRODUCTION

Klebsiella pneumoniae (Kp), a Gram-negative bacterium found in a wide variety of ecological compartments (soil, plants, water), is a common gut colonizer of humans and animals and is an important opportunistic pathogen. Kp is responsible for a broad range of infections, including urinary and respiratory tract infections, liver abscesses, and septicemia. It is a major contributor to the global burden of antimicrobial resistance (AMR) (1), with an increasing number of multidrug-resistant (MDR) Kp infections, including pan-drug-resistant Kp strains (2), being reported. The emergence of multidrug resistance, particularly in hypervirulent, invasive sublineages, has renewed interest in Kp biology and ecology, motivating the development of alternative therapies by which to complement new and existing antimicrobials. One such alternative, namely, phage therapy, relies on the use of virulent bacteriophages (phages), which are viruses that target and kill bacteria (3). Virulent phages may play an important role in controlling Kp populations in different ecological niches, such as soil or the human gut, and they could potentially be harnessed clinically for Kp prophylaxis (decolonization) or therapy.

To infect bacteria, phages rely on their abilities to attach to the bacterial surface, which requires a high specificity toward surface structures. In Kp, the most important external structure is the capsule (K antigen), which is a thick layer of polysaccharides that surrounds the bacterial cell and masks other cell wall structures, such as lipopolysaccharides (LPS) and their corresponding O-antigens. This makes the capsule the primary receptor for most phages that have been isolated against Kp to date (4–12). Except for hypervirulent Kp infections, which are generally associated with a narrow range of K-types, mainly K1 and K2 (13, 14), capsular structure polymorphism is high in the wider Kp population and varies within phylogenetically narrow clonal groups (15–17). Using serological methods, 77 distinct K-types (K1 to K82) have been distinguished (18), and genomic sequencing has thus far uncovered 86 additional capsular polysaccharide synthesis (*cps*) loci (KL-types, KL101 to KL186) (17, 19) which likely synthesize still distinct capsular structures. The distribution of KL-types among clinical isolates of Kp is such that the 6 most frequent KL-types (KL107, KL2, KL24, KL106, KL64, and KL17) represent just 38% of the Kp isolates, and 17 additional KL-types are needed to reach 75% (17).

Another obstacle to Kp phage therapy is the speed at which Kp develops resistance to phages that attach to its capsule, predominantly via the loss of capsule production (9, 20–22). This phage escape mechanism exposes other membrane structures such as LPS, O-antigens, and outer membrane proteins. Interestingly, O-types (eight types that are serologically defined and four others that are putatively defined by genomic analysis) and other surface Kp proteins are less diverse, compared to the capsule, representing potential broad range phage targets. For example, types O1, O2, and O3b represent 80% of human Kp infections (17, 19).

Here, we hypothesized the existence *in natura* of phages that are able to target noncapsulated strains of Kp. Recent studies have shown that, whereas hypercapsulated phenotypes seem to be important for enhanced Kp dissemination in the bloodstream, capsule production is associated with a fitness cost in the gut (23). Moreover, capsule loss seems to be beneficial for epithelial cell invasion and is associated with persistent urinary tract infections (24). Therefore, noncapsulated strains may exist in natural environments and human microbiota and may be preyed upon by their own set of phages.

We also hypothesized that such phages, which are collectively called anti-K^d^ phages here (for anti-capsular deficient, as opposed to anti-K phages that prey on capsulated strains), attach to molecular structures of the cell surface that lie below the capsule and may be leveraged for anti-Klebsiella phage therapy. Such phages have previously been isolated (20, 22), but their broad range of potential and differences from anti-K phages have yet to be studied.

Here, we isolated, characterized, and compared anti-K^d^ phages with anti-K phages *in silico*, *in vitro*, and *in vivo*, including their interactions with bacterial hosts. We show that anti-K^d^ phages have a broad host range, induce a lower rate of emergence of resistance *in vitro*, and have an additive effect in combination with anti-K phages. In particular, we discovered phage mtp5, which can lyse, *in vitro*, nearly all of the noncapsulated Kp strains that were tested. Remarkably, *in vivo*, this anti-K^d^ phage could replicate. Thus, it has lytic capacity in a mouse gut that is colonized with a capsulated Kp strain.

## RESULTS

### Selection of representative capsule-producing and capsule-deficient strains for phage isolation.

The multilocus sequence typing (MLST) of 7,388 publicly available genomes representing the K. pneumoniae species complex (KpSC) revealed a high genetic diversity with more than 1,193 sequence types (STs). However, 7 predominant STs (ST258, ST11, ST15, ST512, ST101, ST307, and ST147) represented 55% of all genomes (Fig. S1; Table S1; Text S1). To reduce bias due to recent clonal expansions or outbreaks, only one strain per ST was retained to analyze the frequency of K-antigen and O-antigen locus types (KL and OL, respectively). KL1, KL64, KL2, KL10, KL30, KL102, KL106, and KL107 were the most prevalent KL-types, whereas OL1, OL2, and OL3/O3a were the predominant OL-types (Fig. S1), together representing 80% of the KpSC genomes.

For the anti-K phage isolation (Table 1; Text S1), seven capsulated strains (Table 1; Table S2) that are representative of the major KL-types were selected as hosts. For anti-K^d^ phage isolation, we selected seven capsule-deficient strains that were derived via mutagenesis in a previous study (25; Table S2) and which represented the three most prevalent OL-types.

**TABLE 1:** Host strains selected for phage isolation^*a*^

ID in this study	Strain ID	SB ID	Species (PhG)	Phenotype	ST	KL-type	OL-type
No. 1Δ	BJ1-GA	SB4496	Klebsiella pneumoniae (Kp1)	Δ*wza*	ST380	mutant (KL2)	O1v1
No. 2Δ	04A025	SB20	Klebsiella pneumoniae (Kp1)	Δ*wza*	ST15	mutant (KL24)	O1v1
No. 3Δ	SA1	SB4021	Klebsiella pneumoniae (Kp1)	Δ*wcaJ*	ST86	mutant (KL2)	O1v1
No. 4Δ	CG43	SB4454	Klebsiella pneumoniae (Kp1)	Δ*wcaJ*	ST86	mutant (KL2)	O1v1
No. 5Δ	NTUH_K2044	SB3928	Klebsiella pneumoniae (Kp1)	Δ*wza*	ST23	mutant (KL1)	O1v2
No. 10Δ	NJST258_2	SB4975	Klebsiella pneumoniae (Kp1)	Δ*wza*	ST258	mutant (KL107)	O2v2
No. 12Δ	342	SB579	Klebsiella variicola subsp. *variicola* (Kp3)	Δ*wza*	ST146	mutant (KL30)	O3/O3a
No. 5	NTUH_K2044	SB3928	Klebsiella pneumoniae (Kp1)	wt	ST23	KL1	O1v2
No. 10	NJST258_2	SB4975	Klebsiella pneumoniae (Kp1)	wt	ST258	KL107	O2v2
No. 31	CIP 52.145	SB3341	Klebsiella pneumoniae (Kp1)	wt	ST66	KL2	O1v2
No. 36	CIP 52.214	SB3245	Klebsiella pneumoniae (Kp1)	wt	ST297	KL10	O1v1
No. 54	NCTC8172	SB504	Klebsiella pneumoniae (Kp1)	wt	ST505	KL64	O1v1
No. 56	1253	SB5521	Klebsiella pneumoniae (Kp1)	wt	ST307	KL102	O2v2
No. 57	899	SB5442	Klebsiella pneumoniae (Kp1)	wt	ST101	KL106	O1v2

a ID, identification; SB, strain bank; PhG, phylogroup; ST, sequence type; KL, capsular locus; O, O-antigen locus; Δ, mutants; wt, wild-type.

### Isolation and genomic characterization of 68 new phages.

We isolated from river and sewage waters and sequenced 68 new phages: 41 anti-K phages and 27 anti-K^d^ phages (Table S3). Here, the prefixes cp (for capsular and phages) and mtp (for mutant and phages) are used to name the anti-K phages and anti-K^d^ phages, respectively. All of the newly isolated phages were categorized as having a virulent lifestyle, based on their genomic sequences. No genes encoding putative virulence factors or antibiotic resistance were carried by any of these phages. A search for putative depolymerases revealed between zero and five depolymerase domains in the genomes of the anti-K phages, whereas the anti-K^d^ phages had zero or one domain (Table S4).

**FIG 1:**
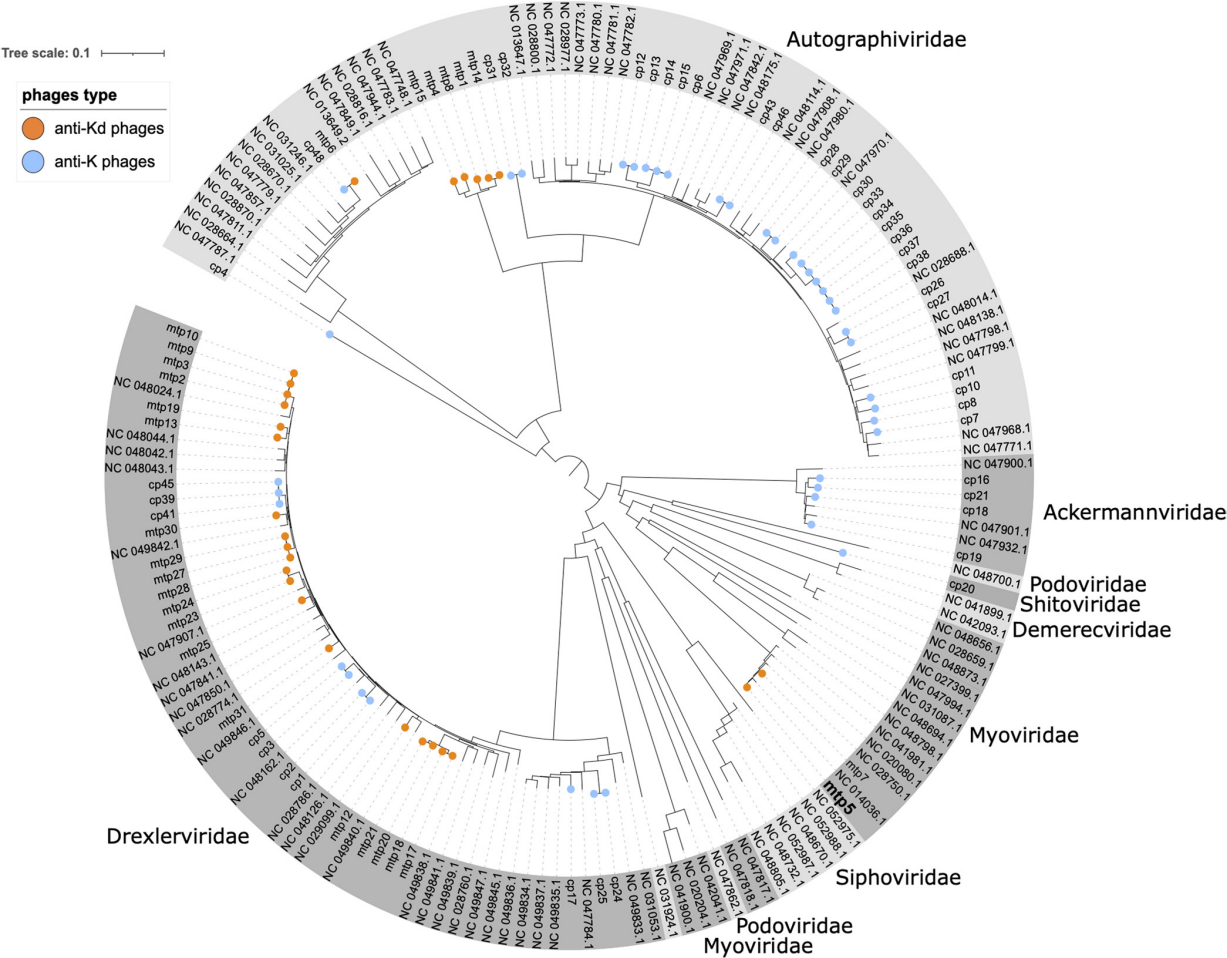
Phage diversity. Genome sequence-based phylogenetic tree of the phages isolated in this study, together with 98 other anti-K. pneumoniae phages (NCBI, March of 2021). Phage families are indicated around the tree. Orange and blue circles at the tips of branches represent the phages isolated in this work: orange, anti-K^d^ phages; blue, anti-K phages. The tree is derived from a k-mer distance matrix (see Materials and Methods).

A phylogenetic analysis of the 68 phages was performed with 98 complete, publicly available anti-Klebsiella phage genomes. The 68 phages were distributed into five phage families: *Autographiviridae*, *Drexlerviridae*, *Myoviridae*, *Ackermannviridae*, and *Schitoviridae* (Fig. 1). Whereas most of the anti-K phages were associated with the *Autographiviridae* family (63%, 26/41), 70% (19/27) of the anti-K^d^ phages belonged to *Drexlerviridae* (Fig. 1; Table S3). High sequence similarity was detected between several of the isolated phages (e.g., cp33, cp34, cp35, cp36, cp37, and cp38 [ANI: 99.9%] or mtp6 and cp48 [ANI: 99.9%]), which indicates that they may be variants of the same phage. However, small nucleotide differences have previously been shown to lead to host range differences (26), as was observed here, when comparing the host range of mtp6 and cp48 (Text S2; Fig. S13). Therefore, we decided to consider them as single phages in the context of this work.

### Anti-K^d^ phages have a broader host range than do anti-K phages.

Based on a panel of 50 KpSC target strains that represented 31 KL-types, the anti-K phages predominantly targeted a single KL-type (Fig. S2; Table S2). Exceptions include: (i) phages cp8 and cp13, which infected the KL10 and KL25 strains; (ii) phage cp34, which infected the KL64 and KL102 strains; and (iii) phage cp16, which targeted the KL35 and KL107 strains. Additionally, phages cp39, cp41, and cp45, which infected capsulated KL106 strains, also infected four out of seven capsule-deficient mutant strains (Fig. S2; Table 1) with the same OL-type (OL1v1). Genomic sequencing revealed the presence of only one depolymerase domain for these three phages, and this domain is homologous to a domain that is present in several anti-K^d^ phages.

**FIG 2:**
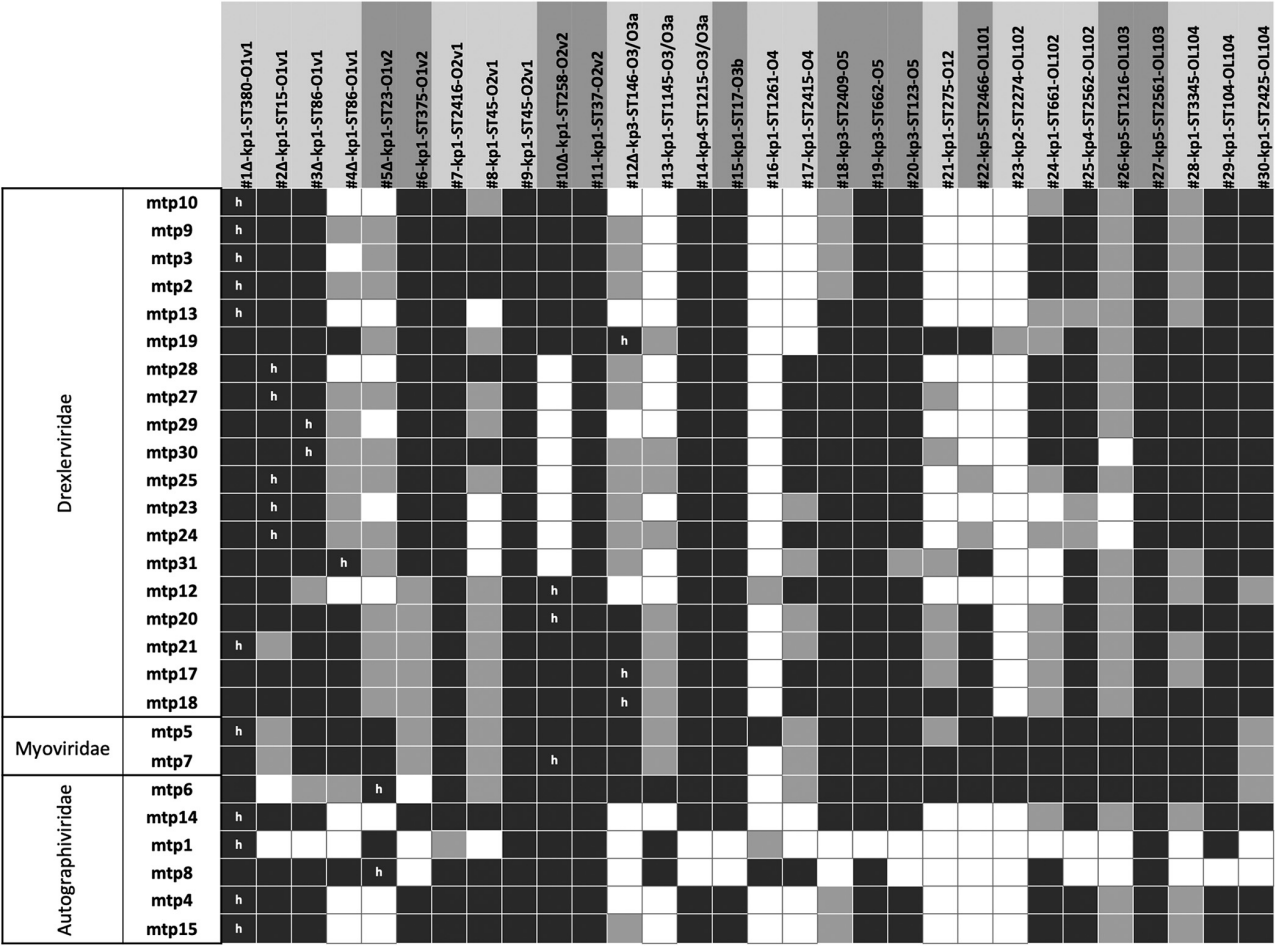
Host range of anti-K^d^ phages. The phages were tested against capsule-deficient strains (*n* = 7, *wza* or *wcaJ* mutants, represented by Δ) and spontaneous nonmucoid culture-derived strains (*n* = 23). Black, complete lysis; light gray, intermediate lysis; white, absence of lysis. “h”: initial host used for phage isolation. The phylogroup, MLST sequence type (ST), and O-antigen type of each isolate is indicated behind the isolate code.

In sharp contrast, the anti-K^d^ phages infected a broader range of strains, with each infecting strains of at least two different OL-types (among the three OL-types of the seven capsule-deficient mutant strains, which have five different KL-types) (Fig. 2; Fig. S3). As expected, the anti-K^d^ phages were inactive against the capsulated strains *in vitro*.

To investigate in greater depth the host range of anti-K^d^ phages, we generated 23 spontaneous non-mucoid clones from KpSC strains belonging to 22 different STs, 18 KL-types, and 11 OL-types (out of the 12 currently known; the missing one, namely, O8, is of low prevalence, representing only 0.03% of the 7,388 genomes analyzed; Table S1) (27, 28). Except for mtp1 and mtp8, which only targeted 10 and 16 strains, respectively, the anti-K^d^ phages infected 20 to 28 strains out of the 30 noncapsulated (capsule-deficient or nonmucoid) strains (Fig. 2). Among these, the broadest host range phages were: (i) mtp5, which infected all of the 30 strains; (ii) mtp7, mtp17, mtp18, mtp20, and mtp21 (28 strains each); and (iii) mtp6, mtp19, and mtp25 (27 strains each) (Fig. 2).

### Characterization of mtp5, which is an anti-K^d^ phage with a broad host range.

Phage mtp5 has a 175.1 kb genome that is composed of 288 CDSs and 1 tRNA gene, and it belongs to the *Slopekvirus* genus within the *Myoviridae* family. Its genomic structure is closely related (identity ranging from 98.43% to 98.98%, with length coverage from 94% to 97%) to that of phage mtp7 (this study) and to other previously described anti-Klebsiella phages: Matisse, Kp27, Kp15, Miro, and PMBT1 (Fig. S4) (29–32). The main genomic difference between mtp5 and these related phages was found in one of the two tail fiber genes (Fig. S4) coding for an L-shaped tail fiber protein. The highest identity of this protein with the above-mentioned phages was with the Miro phage (93.24%). When used as a query against public sequence repositories, this protein showed 95.41% similarity with the L-shaped tail fiber protein of Klebsiella phage Kpn-VAC66 (GenBank accession no. MZ612130.1; a 178 Kb *Myoviridae* phage) (33).

**FIG 3:**
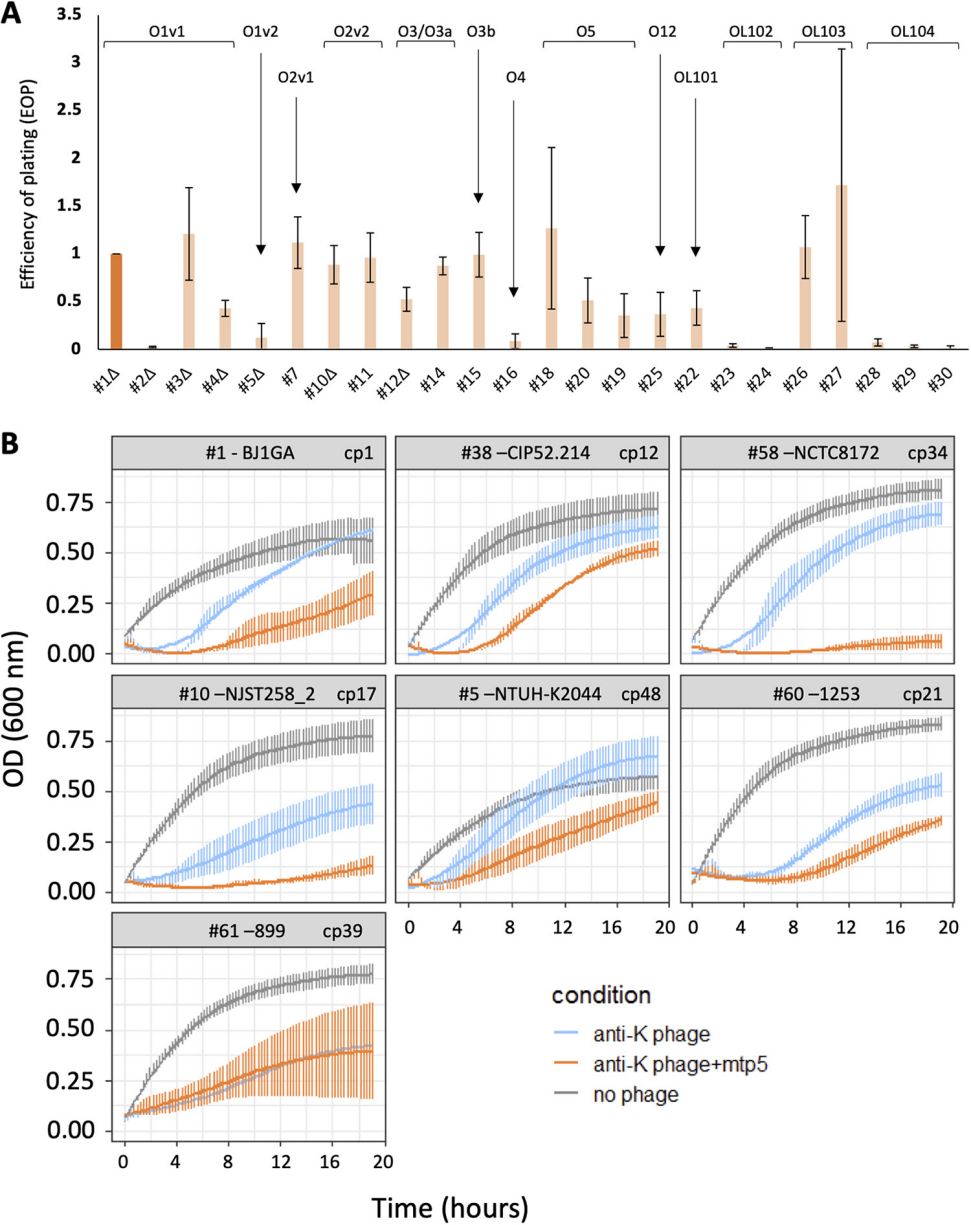
The efficacy of phage mtp5 on spontaneous nonmucoid mutants or in combination with anti-K phages. (A) The efficiency of plating of phage mtp5 on non-mucoid spontaneous mutants. The bacterial strains are ordered by O-type, as indicated, and the EOP was calculated as a ratio of phage titers relative to the host strain, using 1 as the reference value of the host. The darker orange represents the strain used for the mtp5 phage isolation. Only 24 out of 30 noncapsulated strains are represented. For the remaining six strains, we were not able to calculate infection titers, although we could still see lysis with the highest phage concentrations. These conditions might correspond to “lysis from without”, in which high phage concentrations lead to bacterial lysis without actual infection and phage production. (B) The association of phage mtp5 with anti-K phages, assayed against capsulated strains. The growth curves are shown for the indicated Kp strains, either without phage (gray) or with anti-K phages alone (blue) or in cocktail with mtp5 (orange; equal proportions of each). Three to five replicates were performed for each condition. The vertical error bars represent the standard error of the mean (SEM). The phages were added at *t* = 0 at a MOI of 1× 10E−2. The *x* axis scale indicates time in minutes.

Efficiency of plating (EOP) assays confirmed the ability of phage mtp5 to infect almost all noncapsulated strains (Fig. 3A). For some of the strains infected with mtp5, we were unable to assess EOP due to the absence of phage plaques on lower dilutions (Fig. S5). This phenomenon was observed for strains belonging to different OL-types (OL1, OL2, OL3/O3a, OL4, and OL12), yet we were able to assess phage EOP on other strains with the same OL-types. Furthermore, when tested against capsulated strains in combination with anti-K phages (Fig. 3B), phage mtp5 improved the killing (increased lysis) of all tested strains (RBG anti-K+mtp5 versus anti-K, *P* value = 3.38 × 10^−4^), with the lone exception of strain 899 (ST101-KL106-OL1v2) (Fig. 3B).

Other broad host range anti-K^d^ phages were tested in combination with anti-K phages (Fig. S6), and they also showed the increased killing and slow regrowth of escape mutants (see below). These findings are consistent with the observations that were made for mtp5.

### Resistance emerges more slowly against anti-K^d^ phages than against anti-K phages.

We compared the timing of the emergence of subpopulations of bacteria that escape phage predation by anti-K^d^ phages and anti-K phages (Fig. S7A and B). For both categories of phages, the impact of phage predation became visible at 45 to 60 min postinfection. Interestingly, the resurgence of growth was significantly delayed in bacterial cultures that were infected with anti-K^d^ phages, compared to those that were infected with anti-K phages (area under the curve ratios: 0.2 to 0.4 versus 0.4 to 0.6, respectively; *P* value = 0.006, Mann-Whitney test) (Fig. S7C). Simultaneously, we calculated the relative bacterial growth (RBG) at 4 h from cultures with anti-K or anti-K^d^ phages. These were low (from <0 to 0.2, *P* value = 0.4323) for most strains and were not significantly different, indicating the high efficiency of both phage types in controlling their target bacterial populations. However, after 6 h (*P* value = 1.12 × 10^−6^), 8 h (*P* value = 2.20 × 10^−4^), and 10 h (*P* value = 3.33 × 10^−4^), the RBG of the nonsusceptible populations was significantly lower for the noncapsulated strains than for the capsulated strains (Fig. S6D and E), showing the slower emergence of escape mutants among anti-K^d^ phages.

### Genomic changes associated with *in vitro* anti-K and anti-K^d^ phage resistance.

**FIG 4:**
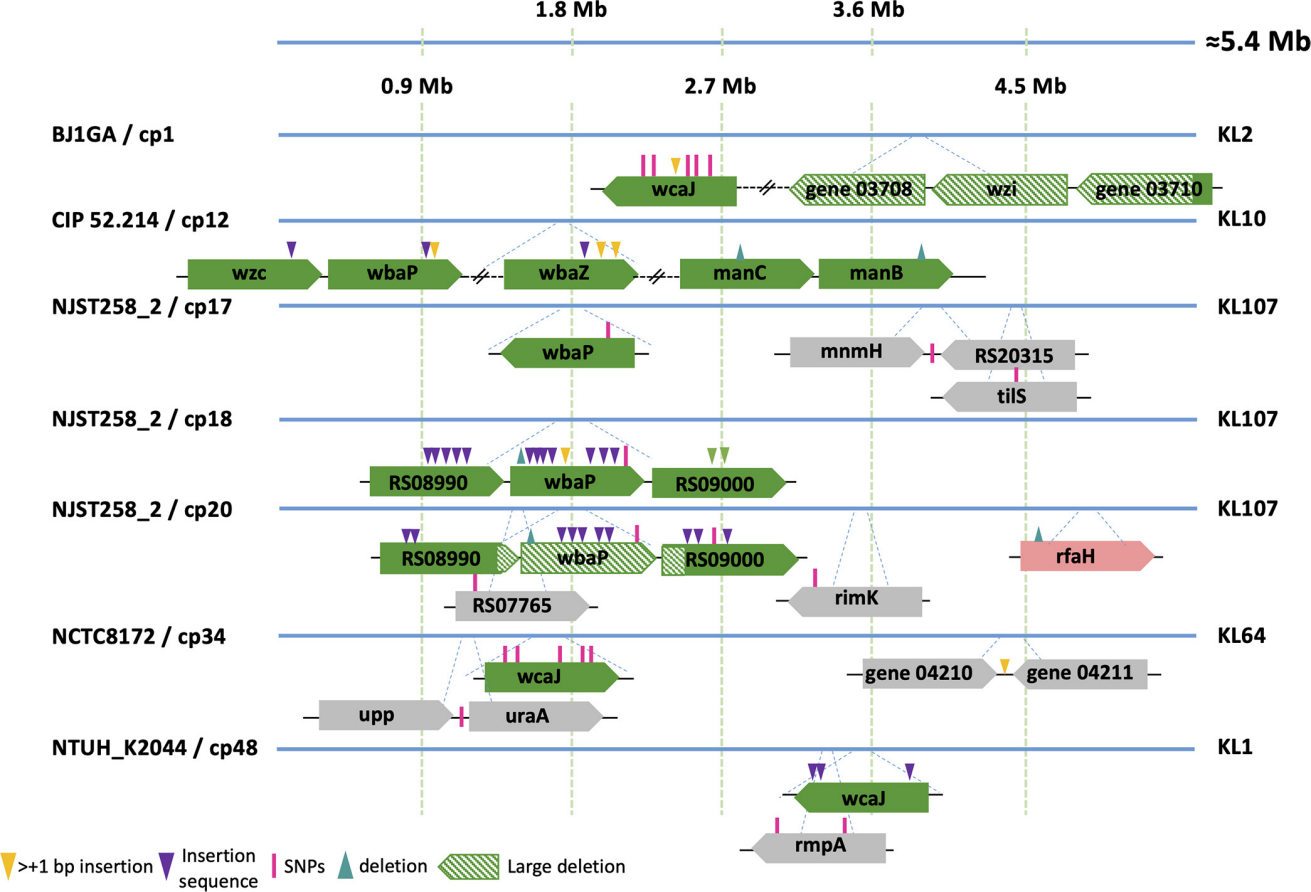
Mutations observed in populations exposed to anti-K phages. Clones were isolated after exposure to anti-K phages. Sequence analysis (see the main text) revealed the mutations that are depicted above. Gene representation colors: green, capsule locus; pink, LPS locus; gray, others. For more details, see Table S5.

Next, we characterized the bacterial genetic changes associated with anti-K and anti-K^d^ phage resistance (Table S5). Populations that were non-susceptible to anti-K phages had mutations in 22 different genes, a majority of which occurred in *cps* cluster genes, mainly *wcaJ* or *wbaP* (Fig. 4; Fig. S8; Table S6). These mutations included single-nucleotide polymorphisms (SNPs), deletions, and disruptions by insertion sequences (IS). The relative frequencies of these events varied, depending on the strain/phage combination. Interestingly, in two cases, *wbaP* was disrupted by a plasmid-borne IS91 family element (Fig. 4; Table S6).

**FIG 5:**
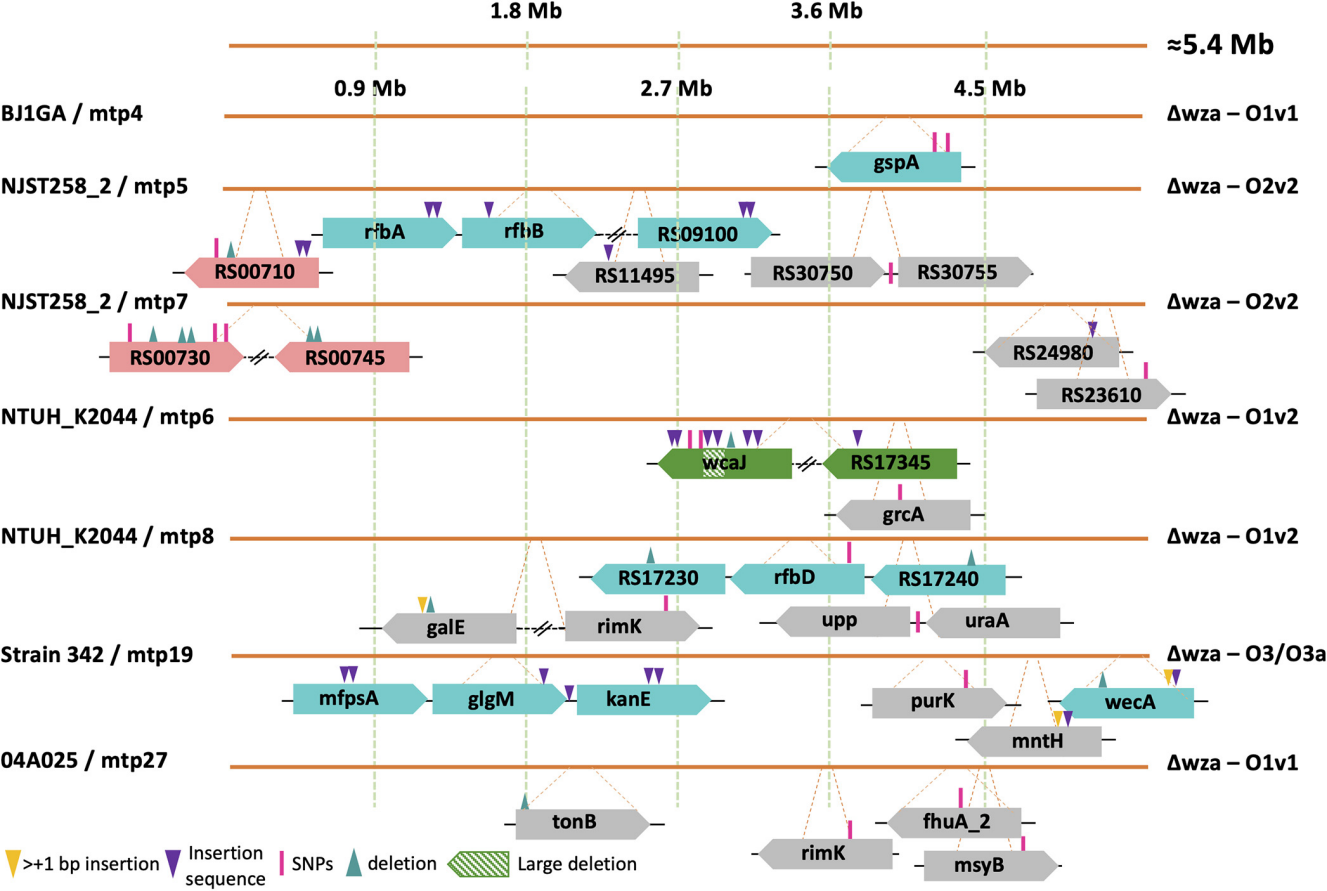
Mutations observed in populations resistant against anti-K^d^ phages. Clones were isolated after exposure to anti-K^d^ phages. Sequence analysis (see main text) revealed the mutations depicted here. Gene representation colors: green, capsule locus; blue: O-antigen locus; pink, LPS locus; gray, others. For more details, see Table S5.

In contrast, resistance against anti-K^d^ phages was associated with more variable events (Fig. 5; Table S6). Overall, mutations in 31 distinct genes from at least 12 different operons were found in anti-K^d^ non-susceptible populations. Half (16/31) of these included mutations in: (i) the O-antigen locus (*gspA* and *rfb* operon, among others) and were observed in four out of the seven strain/phage combinations; (ii) LPS biosynthesis genes, which were mutated in strain NJST258_2Δ*wza* that was exposed to two anti-K^d^ phages; and (iii) surprisingly, in the *cps* locus (*wcaJ* and RS17345 genes), observed in the NTUH_K2044Δ*wza*/mtp6 combination (see additional information in Text S2). The remaining mutations mostly concerned genes coding for transporters (the ferrichrome-iron outer membrane transporter *fhuA*, *tonB* transport system, *uraA* coding for a uracil permease, and *mntH* encoding a divalent metal cation transporter) or genes involved in carbohydrate metabolism (*purK* encoding a formate-dependent phosphoribosylglycinamide formyltransferase and *galE* encoding a UDP-glucose 4-epimerase). We also noted that mutations in the *rimK* (alpha-l-glutamate ligase), *uraA*, and *upp* (uracil phosphoribosyltransferase) genes were associated with both anti-K and anti-K^d^ phage non-susceptible populations, suggesting that they may play a role in the synthesis of both capsular and non-capsular structures.

### Mutations associated with resistance against the broad range anti-K^d^ phage mtp5.

We further explored the genetic changes associated with the resistance to phage mtp5 either alone or in a cocktail with anti-K phages. When mtp5 was used alone, mtp5-non-susceptible populations of BJ1GAΔ*wza* (its original host) emerged after approximately 6.5 h, but no mutations could be detected, as assessed at 18 h postinfection. Therefore, we used strain NJST258_2Δ*wza*, which is also susceptible to mtp5, and found that the population that escaped predation had mutations in several genes that were related to membrane biosynthesis, including the gene RS00710, which encodes a glycosyltransferase that is involved in core LPS biosynthesis (Fig. 5; Table S6).

**FIG 6:**
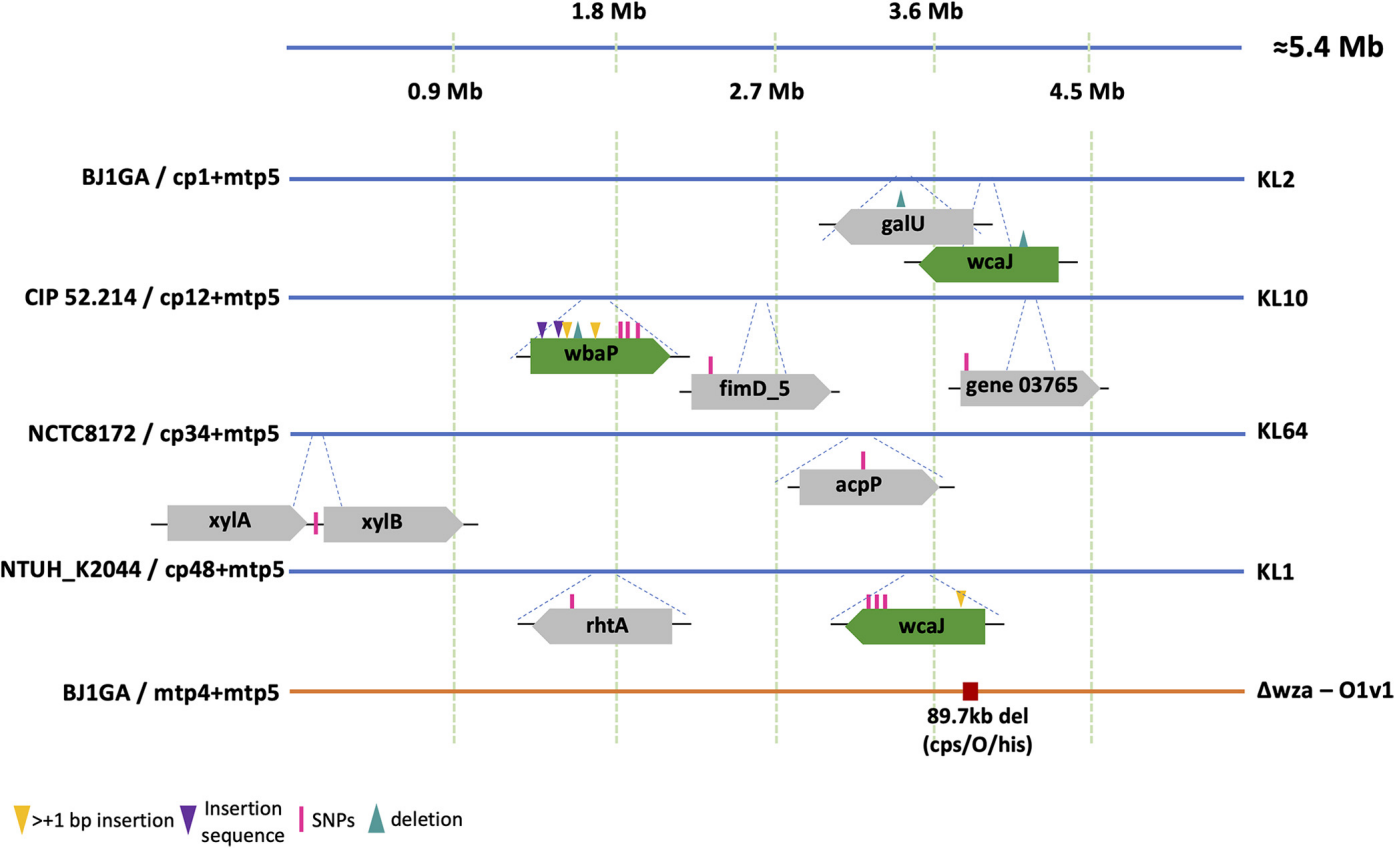
Mutations observed in populations resistant against cocktails with mtp5 phage. Clones were isolated after exposure to cocktails combining anti-K phages and the broad range anti-K^d^ phage mtp5. Sequence analysis revealed the mutations depicted here (for more information, see Table S5). Top (genomes represented by blue lines), capsulated strains; bottom (genome represented by orange line), capsule-deficient strain. Gene representation colors: green, capsule locus; gray, other.

Next, we used four phage cocktails of mtp5 with anti-K phages (cp1, cp12, cp34, and cp48) against capsulated strains (Fig. 6). In total, mutations were detected in eight different genes. Among these were the *cps* initiator glycosyltransferase genes *wcaJ* (BJ1GA/cp1+mtp5 and NTUH_K2044/cp48+mtp5) and *wbaP* (CIP52.214/cp12+mtp5), which were also associated with resistance against anti-K phages alone (Fig. 4). The remaining mutations mainly concerned genes involved in carbohydrate (*galU*, *xylAB*) and lipid (*acpP*) metabolism. For the case of *galU* (encoding an α-d-glucose-1-phosphate uridylyltransferase), a single mutation was detected in a population of BJ1GA that had escaped cp1+mtp5 (this mutation was also detected in BJ1GA against cp1+mtp4). The gene *galU* plays a central role in cell envelope synthesis and was previously associated with resistance to two different anti-Klebsiella phages with impaired growth rates (20). Regarding the gene *acpP*, encoding an acyl carrier protein involved in the biosynthesis of fatty acids and membrane-derived-oligosaccharides in E. coli (34, 35), a non-synonymous point mutation was found in NCTC8172 escaping cp34+mtp5. For this bacteria/phage combination, no genes from the *cps* cluster were affected, suggesting that the observed resistance mutations can be sufficient to prevent infection by the two phages (Fig. 6).

Finally, we tested the phage mtp5 in a cocktail with a second anti-K^d^ phage, namely, mtp4, against the capsule-deficient mutant strain BJ1GAΔ*wza*. The resistant population showed an 89 kb deletion that was comprised of 72 different genes encoding capsule and O-antigen synthesis and for some genes of the core LPS biosynthesis. Additionally, this large deletion also removed the histidine operon as well as the *dnaK* and *pksJ* genes (of the colibactin operon).

### Resistance to other phage cocktails.

Resistance mutations were assessed for three other anti-K/anti-K^d^ phage cocktails (BJ1GA/cp1+mtp4, NJST258_2/cp17+mtp17, and NTUH_K2044/cp48+mtp6) (Text S3; Fig. S9). In general, mutations in the non-susceptible populations were observed in transporters and in genes that are involved in LPS O-locus biosynthesis, similar to what was observed when the anti-K^d^ phages were used alone. Mutations on the *cps* operon (*wcaJ*) were only observed in the NTUH_K2044/cp48+mtp6 experiment, as described above for NTUH_K2044/cp48+mtp5 and NTUH_K2044/mtp6 (Text S2).

### Both anti-K and anti-K^d^ phages can target wild-type K. pneumoniae in the mammalian gut.

**FIG 7:**
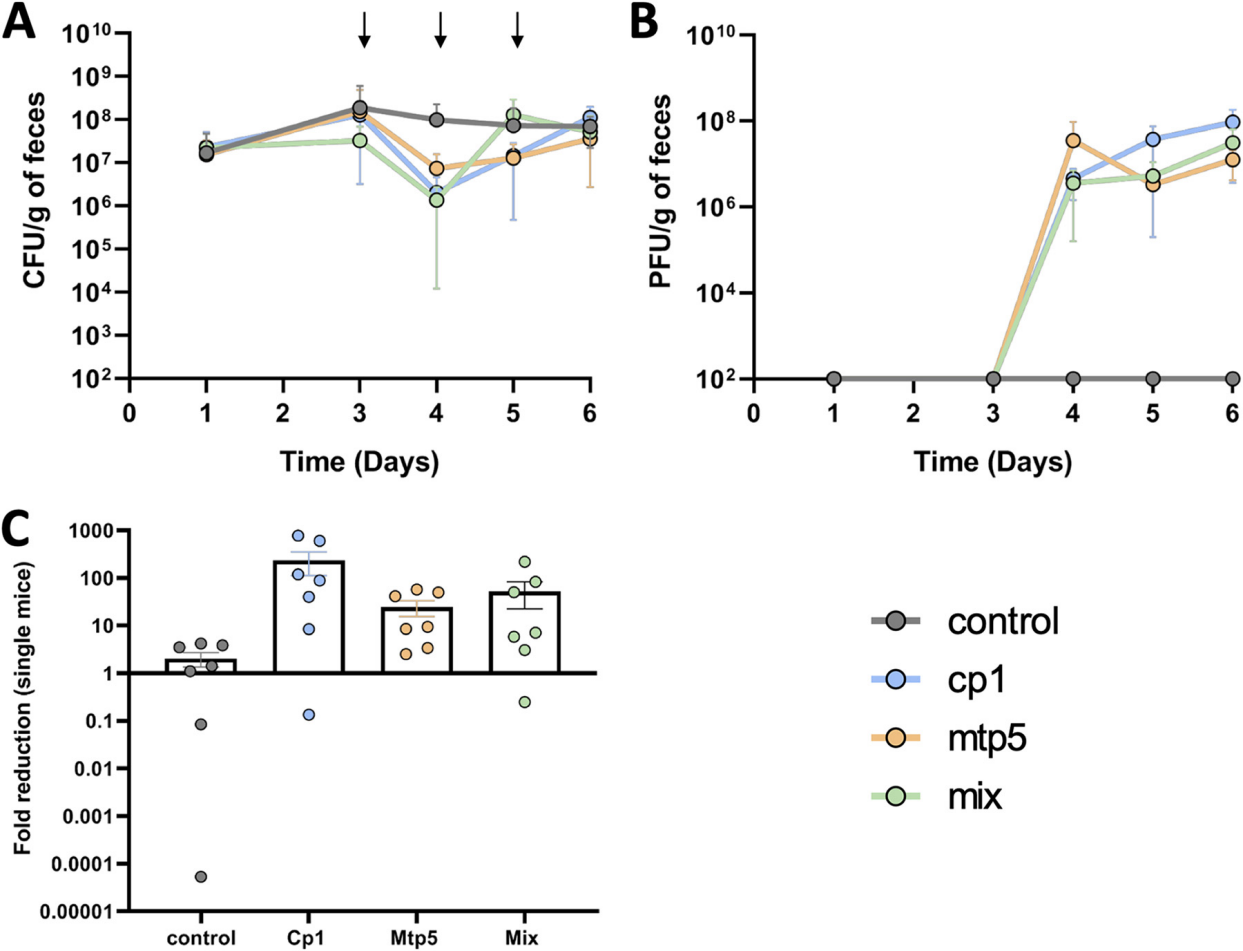
Phage replication and coexistence with K. pneumoniae in the mouse gut. K. pneumoniae BJ1GA-colonized OMM12 mice (*n* = 28), received PBS (gray, *n* = 7) or the phages cp1 and mtp5, either together (mix, green; *n* = 7; 6 × 10^7^ PFU per dose made of the same amount of each phage), or individually (cp1, blue; *n* = 7; mtp5, orange; *n* = 7) via oral gavage on days 3, 4, and 5. (A) Levels of K. pneumoniae BJ1-GA in the feces (the arrows represent the days on which the phage[s] was/were given to the mice). (B) The phage titers from the fecal samples that were reported in panel A. (C) The fold-reduction of the bacterial population levels at one day after the first treatment.

To investigate the abilities of anti-K^d^ phages to infect Kp *in vivo*, we used mtp5 due to its promising host range profile, either alone or in combination with cp1, against the hypercapsulated strain BJ1-GA (the original host of both phages) in mouse gut colonization experiments (oligo-mouse-microbiota mouse model [OMM12]; see Materials and Methods for more details). We observed a stable phage population size for several days, following a single dose of phage, pointing to the replication of both mtp5 and cp1 in the mouse gut, when used either alone or in combination (Fig. S10). However, the observed decrease in fecal Kp abundance was not statistically significant (Fig. S10; Table S7). Hence, we decided to test a recurrent phage challenge within three consecutive days. After repeating the phage administration for 2 more days, a 10-fold to 100-fold decrease in the fecal abundance of the host strain was observed (Fig. 7A and C), albeit again without reaching statistical significance (Table S8).

Kp isolates retrieved from mouse fecal samples varied in their colony morphologies. From the mice receiving phage cp1 and the cocktail (cp1+mtp5), three different colony phenotypes were observed on days four, five, and six: large colonies (hypercapsulated), medium-sized colonies, and small (noncapsulated) colonies (Fig. S11C–E; Table S9). Capsule presence/absence was confirmed via capsule staining assays (Fig. S7), with most of the small colonies retrieved from mice receiving cp1 or the cocktail being encapsulated (with the exception of clone no. 16d5small) and the weak presence of capsule on all of the other clones (with the exception of no. 12d4large). In contrast, similar to the control mice (having received the PBS treatment), we only observed the large and medium colony phenotypes on day four and only the large colonies on days five and six in the mtp5-treated mice (Fig. S11A, B–E; Table S9). The majority of the clones displayed a weak capsule phenotype (capsule staining assays) with only two clones (one from the control and one from the mtp5 group) showing hypercapsule expression (Table S9). These results are consistent with a lack of selection exerted by phage mtp5 for capsule loss.

Next, we investigated whether phage-resistant mutants emerged *in vivo*. One colony of each phenotype was isolated per treatment and per day (25 clones in total) (Fig. S11), and it was tested for resistance to both phages. As expected, for the control and mtp5 groups, all colonies showed susceptibility to phage cp1 but resistance or intermediate resistance to phage mtp5 (as observed in the *in vitro* EOP test; lysis at a high concentration but an absence of plaques at lower dilutions) (Fig. 5; Fig. S11E). In contrast, some colonies that were isolated from the mice treated with phage cp1 or with the mtp5+cp1 cocktail showed cp1 resistance and mtp5 susceptibility (Fig. S11E), suggesting that an escape from cp1 predation via capsule loss caused the susceptibility to mtp5.

To define the mutations associated with these phenotypes, the 25 colonies were sequenced (Table S10). 20 mutations common to all colonies were observed, and these may be associated with adaptation to the gut environment (Table S10). For all of the control colonies, only these mutations were observed. The only exception was a non-synonymous mutation in gene *appA* that coded for a periplasmic phosphoanhydride phosphatase that is normally induced in anoxia, and it was detected on a medium colony from day four from a PBS-treated mouse. The colonies from mice that received phage mtp5 individually showed a similar pattern to that of the control group. Except for one mutation, only common mutations were observed. Indeed, no resistance mutations were expected if the capsulated strain given at gavage was recovered in the feces, with no genomic change affecting capsule expression during mouse gut passage. Interestingly, the only exception was one large phenotype colony that was isolated from feces from day six, which presented a mutation on the gene *galR*, which is the transcriptional repressor of the operon that is involved in the transport and catabolism of d-galactose.

In contrast, colonies from the mice that received anti-K phage cp1 showed the presence of several mutations that were distributed in all colonies isolated at days five and six, except for the hypermucoid ones (large phenotype). As observed *in vitro* in resistant populations against anti-K phages, most of these mutations were observed on the genes *wcaJ*, *kanF*, and *galU*. These changes may explain the smaller and nonmucoid aspects of the resistant colonies and are compatible with capsule loss as a resistance mechanism.

Finally, in the colonies from the mice that received the two-phage cocktail, we detected mutations on the first day after treatment (day four) as well as on days five and six. The target genes included the gene *gspA* from the type II secretion system operon, the gene *ompC* for the outer membrane protein C (a known phage receptor in E. coli), and the gene SB4496_Kp1_03710, which belongs to the capsule synthesis operon (Table S10). Therefore, when used in combination *in vivo*, cp1 and mtp5 appeared to induce resistance mutations that were previously observed *in vitro* against anti-K and anti-K^d^ phages.

## DISCUSSION

In this work, we developed an original approach to isolate phages against noncapsulated Kp mutants (here, called anti-K^d^ phages), based on the hypothesis that they target conserved structures that lie below the capsule. Consistent with our initial hypothesis, phylogenetically diverse anti-K^d^ phages were isolated, and these were able to infect a broad range of noncapsulated Kp strains. The existence of such phages indicates that they have access to target bacterial populations in the natural environment, and their broad spectrum *in vitro* suggests that they represent ecologically more generalist phages than those targeting the capsule. The most prominent example was phage mtp5, which may display the widest host range of Kp phages isolated thus far. This phage targeted most of the noncapsulated strains that were tested and, when combined with anti-K phages, enhanced the population reduction effect against almost all of the capsulated Kp strains. In contrast, a marked host specificity was observed for the anti-K phages, which were typically only able to target a unique KL-type. These findings are consistent with a large body of previous work. In a recent study, phages showing a capsule-independent mode of entry also exhibited a much broader host range than phages relying on the capsule for entry (12). From a therapeutic perspective, capsule-specialist phages may represent useful tools against certain specific Kp infections, for example, in the case of liver abscesses, which are localized infections that are mainly associated with the K1 and K2 capsular types (13, 14). However, for most other types of infections, the capsular diversity of Kp is high (17) and represents a major hurdle to phage therapy.

Exceptions to the “one anti-K phage, one capsular type” rule were observed among a minority of the anti-K phages studied here, including cp8, cp10, cp11, cp12, and cp13, all of which were isolated from a KL10 host strain and were also able to infect KL25 strains. A structural analysis and comparison of the KL types that were infected by the same phage did not reveal obvious structural commonalities in the CPS region (data not shown). This suggests that the ability of these phages to infect strains with different KL types may be explained by the presence of different depolymerase functional domains in the tail fibers of these phages (Table S4). Depolymerase domains confer to virulent anti-Klebsiella phages the ability to lyse specific exopolysaccharides. Anti-Kp phages carrying multiple depolymerase domains have been described previously, but the broadest-range phage was able to infect only 11 different KL-types (11, 36, 37), which is insufficient in the face of the approximately 160 capsular types that have been described or inferred via genomics thus far (19).

In addition to their broad host range, anti-K^d^ phages appear to induce a lower rate of emergence of escape mutants, compared to the anti-K phages, and this is even more so when they are used in a cocktail. The delayed emergence of anti-phage resistance may represent an important benefit for therapeutic use. In Klebsiella, the emergence of phage resistance *in vitro* or during therapy is an important issue for anti-K phages, with capsular loss (mainly via mutations or disruptions mediated by insertion sequences on the glycosyltransferase initiators of capsule production, namely, *wcaJ* and *wbaP*) being the most common resistance mechanism, as shown here and previously (20, 38, 39).

Resistance to anti-K^d^ phages was associated with a variety of mutations that have been inferred to disrupt several surface structures in phage-resistant bacterial populations, suggesting that a wide set of receptors can be used by anti-K^d^ phages. Among these structural elements, the LPS core was often affected, as was observed for mtp5 resistance alone or in a cocktail, where mutations in several genes that were involved in the production of the initial building blocks of LPS biosynthesis were uncovered. The conservation of LPS in Kp (40) is consistent with the broad host range exhibited by the anti-K^d^ phages that induced LPS core mutations.

The loss of particular membrane proteins or components of the core LPS may incur a high fitness cost, which could delay the development of anti-K^d^ phage resistance by necessitating physiological adjustments to compensate for important changes to the cell membrane. Whereas the capsule is costly to produce and is dispensable or even deleterious in some environments (24, 41), the structures targeted by anti-K^d^ phages may correspond to more conserved and essential components of the bacterium. This study calls for the future characterization of anti-K^d^ phage receptors, the nature of which will have implications for mechanistic insights into phage infection, the host range, and the fitness costs of resistance.

A comprehensive knowledge of whether capsule production is systematic, abundant, and stable *in vivo* is essential to better understand Klebsiella pathogenicity and perspectives for phage therapy. A lack of capsule expression by Kp populations in colonization or infection sites may have critical impacts on anti-K phage efficacy *in vivo*, thereby supporting the relevance of our proposed anti-K^d^ phage strategy. For example, capsule loss may be beneficial for epithelial cell invasion and is associated with persistent urinary tract infections (24), thereby supporting the exploration of anti-K^d^ phages for the treatment of multidrug-resistant Kp urinary tract infections. In particular, the complexity of the gut environment leads to multiple ecological interactions, and capsule expression may be associated with variable fitness costs (23, 42). Our capsulated Kp strain decolonization experiments showed the replication of the anti-K phage, as expected, as well as that of the anti-K^d^ phage mtp5 in the mouse gut, with a transient reduction of the bacterial load. Similar coexistence dynamics of bacterial hosts and their virulent phage populations in the mouse gut have previously been reported (43–47). Importantly, the replication of the anti-K^d^ phage mtp5, when administered alone, suggests that capsule expression is not constitutive in Kp populations during gut colonization, even in the absence of selection by anti-K phages. Given the fitness costs related to the capsule, its expression may be nonuniform along the gastrointestinal tract, over time, or in specific subniches (23, 44, 48). Whereas the cp1 anti-K phage seems to play an important selective role *in vivo*, as it induces the emergence of noncapsulated resistant morphotypes, this was not the case for the phage mtp5 when used alone, even though this phage maintained a stable population size. This suggests a transient lack of capsule expression in a minority of the Kp population in the gut, which enables phage replication while having no effect on the capsulated population.

In turn, when both phages were used in combination, the observation of small colony phenotypes in mouse feces (Fig. S11) is compatible with noncapsulated Kp variants evolving to escape anti-K phages in the gut, thereby generating novel hosts for the anti-K^d^ phage with consequent infection and replication, as was observed here for mtp5. In agreement with this, we observed that fast-emerging mutations in the colonies that were isolated from these mice affected some of the genes that were observed in *in vitro* resistance against anti-K and anti-K^d^ phages.

Heterogeneity in capsule production within Kp populations implies that the simultaneous use of anti-K^d^ and anti-K phages may be necessary to achieve population depletion. Furthermore, capsule-deficient strains of Kp are more prone to conjugation, and plasmids are acquired at higher rates by noncapsulated isolates (49). Hence, an anti-noncapsulated Kp strategy may also contribute to the reduction of the transmission of plasmid-borne virulence and antimicrobial resistance.

In summary, we showed that anti-K^d^ phages, which we defined as anti-Klebsiella phages that are virulent for capsule-deficient mutants, can be isolated from environmental water sources and combined several interesting properties that offer promise for phage therapy: a broad host range across the great diversity of Kp species sublineages, the slower emergence of phage resistance, and an additive effect in combination with anti-K phages. Some of the anti-K^d^ phages uncovered in this work, such as mtp5, had a complete or nearly complete host range, as assessed based on O-antigen diversity. Our *in vivo* experiments further showed that such anti-K^d^ phages may represent a useful complement to anti-K phages for successful Kp decolonization or therapy. Thus, this work provides realistic avenues for the development of Kp phage therapies that circumvent the narrow spectrum limitations of anti-K phages. Considering the importance of the capsule in Kp pathogenicity and the potential role of anti-K^d^ phages in anti-Klebsiella phage therapy, more work is needed to address the pivotal questions of where, when, and how much the capsule is expressed during Kp colonization or infection.

## MATERIALS AND METHODS

### Bacterial strains and phages.

The bacterial strains used for the phage isolation and host range assays are listed in Table S2. The strains were routinely cultured in lysogeny broth (LB), or on LB agar or Simmons Citrate agar with Inositol (SCAI) plates at 37°C.

The phages that were isolated in this study are described in Table S3. The phages were amplified in exponential growing cultures of the respective host strain for approximately 4 to 5 h at 37°C in aerated LB at 150 rpm. The cell lysate supernatants containing amplified phages were 0.22 μm filter sterilized and stored at 4°C.

### Genomic background and surface structure analysis of publicly available genomes of the K. pneumoniae species complex for phage host strain selection.

In total, 7,388 genomic sequences that were previously collated from the NCBI GenBank database in March of 2019 and that belonged to the K. pneumoniae species complex (KpSC) were included (50, 51). Comparative genomic analyses were performed using Kleborate v1.0 (17), which is a tool that was designed for the genotyping (species assignment, classical 7-gene MLST, KL-antigen and O antigen locus (OL) type, AMR and virulence determinants) of KpSC (17).

Based on the genotyping information that was collected, we selected a total of 14 KpSC strains for phage isolation (Table 1). These included seven capsulated strains (all K. pneumoniae, Kp1) and seven capsule-deficient mutants (six Kp1 and one *K. variicola*, Kp3) (25) as hosts for the isolation of anti-K phages and anti-K^d^ phages, respectively.

### Phage isolation and host range tests.

Phage isolation was performed using water from the River Seine and from sewage, as previously described (44). The isolation was performed not only with wild-type strains but also with clean acapsular mutants (see bacterial strains). To increase the probability of isolating broader host range phages, the isolation was performed in single strain and multiple strain cultures. Host range tests were performed as follows: 3 μL of phosphate-buffered saline (PBS)-diluted phage solutions (0.22 μm filter sterilized crude lysates adjusted to 10^7^ PFU/mL) that were deposited side by side on the lawn of each tested bacterium on square LB agar plates. The plates were incubated at 37°C overnight (approximately 18 h).

Isolated phages were tested against 50 different KpSC strains, including 43 capsulated strains representing 32 different KL-types and the 7 capsule-deficient mutants (25) (Table S2) as well as against 16 strains from other *Enterobacteriaceae* species (9 Escherichia coli, 6 Salmonella spp. and 1 *Citrobacter* spp.). Additionally, the anti-K^d^ phages were also tested against 23 KpSC nonmucoid (presumably capsule-deficient) clones that were generated as explained below (Table S2).

### Generation of nonmucoid KpSC strains (capsule-deficient clones).

Following Chiarelli et al. (52), 23 capsulated KpSC strains were streaked on tryptic soy agar (TSA) and incubated at 37°C for 24 h. Then, the TSA plates were scanned for nonmucoid (NM) sectors (Fig. S12) at 25°C until NM sectors were observed. The sectors were then isolated, plated, kept at 37°C for another 24 h, and then left at 25°C to verify whether all of the colonies were homogenous. If so, two further steps of purification were performed before storage at −80°C (Table S3).

### Capsule staining assays.

Capsule staining was performed by placing a 5 μL drop of an overnight culture of the bacteria to be tested with a 3 μL drop of nigrosine (Chinese ink) on a slide. The slide was then observed under a microscope (100×, oil immersion) for the presence/absence of capsule.

### Phage efficiency of plating (EOP) tests.

For each phage, the efficiency of plating (EOP) was calculated as the ratio of the number of plaques that were formed by the phage on each strain tested and the number of plaques that were formed on the original isolation strain. 3 to 4 independent replicates were performed using bacterial cultures that were grown to an optical density (OD) of approximately 0.2 at 600 nm and spread on LB plates onto which phage dilutions were spotted. The plates were incubated at 37°C overnight.

### Phage genome sequencing and analysis.

Sterile phage lysates (obtained by a 5 h phage amplification that was followed by centrifugation [10 min, 5,000 rpm] and 0.22 μm filter sterilization) were treated with DNase (120 U) and RNase (240 mg/mL) for 30 min at 37°C before the addition of EDTA (20 mM). The lysates were then treated with proteinase K (100 mg/mL) and SDS (0.5%) at 55°C for 30 min. The DNA was extracted via a phenol-chloroform protocol that was modified from Pickard, 2009 (53). The genomic DNA libraries were prepared with a TruSeq DNA PCR-Free Sample Preparation Kit (Illumina Inc., San Diego, USA), and 2 × 150 paired-end sequencing was performed using NextSeq 500/550 Illumina technology (Illumina, San Diego, USA). The quality of the reads was checked with fastqc v0.8.5 (https://www.bioinformatics.babraham.ac.uk/projects/fastqc/), and the reads were cleaned using the fqcleaner pipeline from Galaxy-Institut Pasteur (https://gitlab.pasteur.fr/GIPhy/fqCleanER). The *de novo* assembly was performed by either using SPAdes (3.11.0) (54) or using a workflow implemented in Galaxy-Institut Pasteur that used clc_assembler v4.4.2 and clc_mapper v4.4.2 (CLC Bio, Qiagen) when necessary. The phage termini were determined by PhageTerm 2.0.1 (55), and the annotations were performed using PATRIC RASTtk (56, 57). The phage lifestyles were accessed using BACPHLIP, which is a Python library for predicting phage lifestyle based on genome sequence (58). The presence of genes coding for putative virulence factors or antibiotic resistance was investigated using Kleborate, Resfinder, BIGSdb Pasteur Klebsiella, and/or a virulence genes database (VDFB) (17, 59–61).

Phylogenetic trees were generated using JolyTree v2.0 using the whole phage genome data (MASH based; parameters: sketch size of 100,000, probability of observing a random k-mer of 0.00001, and k-mer size of 15) (62). The phylogenetic trees were visualized using iTol (63). 98 publicly available genomes that were deposited on the RefSeq NCBI database with Kp (taxid: 573) stated as the host (March of 2021), were used for comparative analyses. The average nucleotide identity (ANI) between similar phages was calculated using OGRI (https://gitlab.pasteur.fr/GIPhy/OGRI). The protein structures of the phages cp48 and mtp6 were predicted using AlphaFold (64).

### Analyses of depolymerases.

To detect putative depolymerases in our newly isolated phages, we performed an initial HMMER (v3.3) comparative analysis using 14 HMM profiles that are associated with bacteriophage-encoded depolymerases (25) (filtered by the E value of the best domain maximum 10^−3^). A BLASTP (v2.6.0, default parameters) search, filtering by E value (maximum 10^−5^) and identity (30%), was also performed for specific protein sequences that were described and validated for broad range Klebsiella phages had been described in the literature (36) (Table S4).

### Growth curves and lysis kinetics analysis.

To record the phage replication and bacterial lysis, an overnight culture of the respective bacterial strain was diluted in LB Lennox media and grown to an OD at 600 nm of approximately 0.2, from which 140 μL were distributed on a 96-well plate (Microtest 96 plates, Falcon). Afterwards, 10 μL of sterile phage lysates that had previously been diluted in PBS were added to obtain a multiplicity of infection (MOI) of 1 × 10^−2^ in each well. The plates were incubated in a microplate reader at 37°C with a shaking step of 30 s before the automatic recording of the OD at 600 nm every 15 min over 18 to 20 h (Tecan microplate reader). The area under the curve (AUC) was calculated in R using the trapz function from the pracma package (v2.3.3). The relative bacterial growth (RBG) was calculated using the following equation: RBG = (Abs600 [t = 4 h] − Abs600 [t = 0 h]) bp / (Abs600 [t = 4 h] − Abs600 [t = 0 h]) b, in which Abs600 represents the absorbance at 600 nm of the bacterial cultures, b represents that for bacteria only (control cultures), bp represents that for bacteria with phage (infected cultures), and t represents the time in hours (h). The RBG values were calculated at 4, 6, 8 and 10 h (22).

### Isolating and testing putative phage-resistant clones.

After 20 h of growth, cultures were plated in LB Lennox agar to select possible phage resistant clones. After approximately 18 h at 37°C, colonies were counted and checked for phenotypic differences. In total, 24 colonies from the control cultures and 50 colonies from the infected cultures were isolated and grown in 96-well plates with LB Lennox plus 16% glycerol for 24 h at 37°C. Then, they were stored at −80°C. Afterwards, a fresh overnight culture of each clone (i.e., each isolated colony) was grown at 37°C and refreshed in LB liquid media the following day, and it was grown at 37°C to an OD of approximately 0.5 (600 nm). The phage infectivity was further tested using a double spot test (6 μL bacterial culture + 3 μL phage) in order to scan for resistance.

Subsequently, a fresh culture of 1 mL of each clone (i.e., each isolated colony) was grown for 24 h at 37°C. Then, all of the cultures were pooled and centrifuged. This pelleted population was then diluted in 1 mL of PBS. 500 μL of the sample were used for DNA extraction using a Maxwell Cell Tissue Kit (Promega) and were subjected to Illumina sequencing, as described above.

For some cultures, the detection of a small colony variant phenotype led to isolation difficulties. In such cases, DNA extraction was performed directly from the colonies that were collected from the plate, without a previous resistance test (Table S9).

The initial phenotypic observations (colonies on LB plates) regarding the anti-K phages showed an apparent loss of capsule within the population at 20 h postinfection (to control for capsule presence, colonies were tested from samples in the presence and absence of phages). Colonies with the different phenotypes were counted before isolation for the resistance assay and subsequent DNA extraction and sequencing (Table S8).

### Animals and ethics.

Oligo-MM12 C57BL/6NTac mice were bred in isolators (Getinge) in the germfree facility at the Helmholtz Center for Infection Research. The experiments were performed in gender-matched and age-matched animals, specifically, female and male mice with an age of 8 to 12 weeks. Sterilized food and water were provided *ad libitum*. The mice were kept under strict 12-h light cycles (lights on at 7:00 a.m. and off at 7:00 p.m.), and they were housed in groups of 3 to 4 mice per cage. All mice were euthanized via asphyxiation with CO_2_ and cervical dislocation.

All animal experiments were performed in agreement with the guidelines of the Helmholtz Center for Infection Research, Braunschweig, Germany; the National Animal Protection Law [Tierschutzgesetz (TierSchG)] and Animal Experiment Regulations [Tierschutz-Versuchstierverordnung (TierSchVersV)], and the recommendations of the Federation of European Laboratory Animal Science Association (FELASA). The study was approved by the Lower Saxony State Office for Nature, Environment and Consumer Protection (LAVES), Oldenburg, Lower Saxony, Germany; permit No. 33.8-42502-04-20/3564.

### Murine model of K. pneumoniae colonization.

*In vivo* colonization assays were performed using the OligoMM (14) mouse model. This mouse model harbors a consortium of 12 different bacterial strains in the gut (65). Two independent experiments with repeated phage administration were performed with *n* = 3 to 4 mice per group. On day 0, the mice were orally colonized with the Kp bacterial strain BJ1-GA (SB4496). The initial inoculum was prepared by culturing bacteria overnight at 37°C in LB broth with 25 mg/mL chloramphenicol. Subsequently, the culture was diluted 1:25 in fresh LB medium and subcultured for 4 h at 37°C. The bacteria were resuspended in 10 mL of PBS and adjusted to 5 × 10^8^ CFU/mL. The mice were orally inoculated with 200 μL of bacterial suspension. On day 3 or on days 3, 4 and 5, the mice received the two phages alone (adjusted to 10^8^ PFU) or in combination (1:1 mixture of each phage in 200 μL of PBS) once daily via oral gavage.

The level of phages was assessed via serial dilutions in PBS and spotting on LB plates with an overlay of strain BJ1-GA. The weights of the mice were monitored during the course of the experiment, and feces were collected at different time points after colonization (days 1, 3, 4, 5, and 6). Subsequently, fecal samples were diluted in 1 mL PBS and homogenized via bead-beating with 1 mm zirconia/silica beads twice for 25 s using a Mini-Beadbeater-96 (BioSpec). To determine the CFU, serial dilutions of homogenized samples were plated on LB plates with 25 mg/mL chloramphenicol. To determine the PFU, serial dilutions of homogenized samples were plated on LB plates overlaid with strain BJ1-GA. The plates were incubated at 37°C overnight before counting. The CFU of strain BJ1-GA and the PFU of the phages were calculated after normalization to the weight of the feces. For the isolation of clones with different phenotypes, the fecal samples of one mouse per group were plated on SCAI medium agar, and one clone of each different phenotype was isolated in LB Lennox agar medium. This was followed by testing for susceptibility to the phages cp1 and mtp5 as well as sequencing.

### Statistical analysis.

For the *in vitro* growth curves and lysis kinetics, statistical analyses were performed in R, using a Mann-Whitney test to compare the AUC and RBG values. The statistical analyses of the bacterial levels in the *in vivo* mouse experiments were carried out using the lme4, lmerTest, and car packages in R (66–68). The CFU numbers were log_10_-transformed prior to analysis. In each experiment, four groups of mice were considered: three groups that were exposed to the different phages (cp1, mtp5, cocktail) and an unexposed control group. The impact of the phages was assessed based on the abundance of phages (log-PFU). Given the nonlinearity of the responses, the day at which a measure was performed was considered to be a categorical variable. Linear mixed-models were used to account for random experimental effects (i.e., individuals and experimental effects). Overall effects were assessed via an analysis of variance (ANOVA), and *post hoc* Tukey’s comparisons were performed by using the lsmeans package in R (69). For all of the analyses, a *P* value of <0.05 was considered to be indicative of a statistically significant result.

### Data availability.

The reads can be found in the NCBI SRA public archives under BioProject accession no. PRJEB55170. The genome assemblies of the phages that were isolated in this study have been deposited into the European Nucleotide Archive under the BioProject accession no. PRJEB55170.
