# Supplementary material for: Phages against Noncapsulated Klebsiella pneumoniae: Broader Host range, Slower Resistance
Source: Microbiol Spectr. 2023 Jun 20;11(4):e04812-22. doi: 10.1128/spectrum.04812-22 (PMC10433977; doi:10.1128/spectrum.04812-22)
Supplement: Supplemental file 1 — Supplemental material. Download spectrum.04812-22-s0001.pdf, PDF file, 6.3 MB [file spectrum.04812-22-s0001.pdf]

Supplementary Material

Phages against non-capsulated *Klebsiella pneumoniae*: broader host range, slower resistance

Authors

Marta Lourenço[1], Lisa Osbelt[2], Virginie Passet[1], François Gravey[3], Daniela Megrian[4], Till Strowig[2], Carla Rodrigues[1], Sylvain Brisse[1]

1 Institut Pasteur, Université Paris Cité, Biodiversity and Epidemiology of Bacterial Pathogens, Paris, France

2 Department of Microbial Immune Regulation, Helmholtz Center for Infection Research, Braunschweig, Germany; ESF International Graduate School on Analysis, Imaging and Modelling of Neuronal and Inflammatory Processes, Otto-Von-Guericke University, Magdeburg, Germany.

3 Dynamycure Inserm UM1311 Normandie Univ, UNICAEN, UNIROUEN, 14000 Caen, France

4 Unité de Microbiologie Struturale, Institut Pasteur, CNRS UMR 3528, Université Paris Cité, F-75015, Paris, France

Supporting information

Supplementary text 1 - Strain selection for phage isolation

Supplementary text 2- Anti-K phage cp48 and anti-K^d phage mtp6 may share the same receptor

Supplementary tables:

Table S1. Dataset of 7,388 public *K. pneumoniae* species complex genomes

Table S2. Collection of bacterial strains used in this study

Table S3. Main characteristics of the phages isolated in this study

Table S4. Depolymerase analysis

Table S5. Percentage of resistant clones *in vitro*

Table S6. Analysis of the mutations acquired after phage infection in vitro (Breseq analysis)

Table S7. Statistics - 1 day phage treatment

Table S8. Statistics - 3 days phage treatment

Table S9. *In viv*o resistance phenotypes

Table S10. Analysis of the mutations acquired after phage infection *in vivo* (Breseq analysis)

Table S11. Lysis Kinetics data relative to cocktails including mtp5 and anti-K phages

Please see the separate Excel file for access to the supplementary tables.

Supplementary figures:

Figure S1. Frequency of *Klebsiella pneumoniae* genomes broken down by ST, KL or OL characteristics.

Figure S2. Host range of anti-K phages.

Figure S3. Lysis kinetics of phage mtp5 on the 7 capsule-deficient mutant (Δ*wza* or Δ*wcaJ*) strains.

Figure S4. Anti-Kd phages mtp5 and mtp7 genomic similarities with closest anti-Klebsiella phages.

Figure S5. Example of titration of phage mtp5 on the generated spontaneous non-mucoid clones.

Figure S6. Cocktails of phages mtp4, mtp6 and mtp7 with anti-capsule phages.

Figure S7. Subpopulations of bacteria non-susceptible to phages emerge slowly when using anti-K^d phages against non-capsulated strains.

Figure S8. Resistance to anti-K phages leads to smaller (presumably less capsulated) colonies**.**

Figure S9. Mutations observed in populations exposed to phage cocktails that combine anti-K phages and anti-K^d phages mtp4, mtp6 and mtp7.

Figure S10. Phage mtp5 does not infect wild-type strain BJ1GA *in vitro* but does *in vivo.*

Figure S11. Isolation of different phenotypes from the feces of mice during phage treatment**.**

Figure S12. Example of non-mucoid sectors.

Figure S13. Comparison of phages mtp6 and cp48 for their genomic and proteic structures and infection phenotype.

Supplementary text 1:

Strain selection for phage isolation

Based on our population genomics analysis (**Figure S1**), we first selected 7 *K. pneumoniae* strains as hosts for anti-capsulated strain phage isolation:

- *K. pneumoniae* NJST258_2 (SB4975), a representative of the ST258-KL107 group, one of the most widespread lineages in clinical settings and linked to nosocomial infections and outbreaks[1]
- *K. pneumoniae* NCTC8172 (SB504), a ST505 harbouring the *cps* locus (KL) structure type 64, one of the most disseminated KL-types, which has been extensively associated with two widespread carbapenemase-producing sequence types (ST), ST147 and ST11 [2,3].
- strains SB5442 and SB5521, representing two successful MDR ST-KL (KL: capsule locus) combinations in clinical settings, ST101-KL106 and ST307-KL102, respectively [4,5].
- *K. pneumoniae* strain CIP52.214 (SB3245), a ST297-KL10, was selected as it represented one of the most prevalent KL-types.
- two hypervirulent *K. pneumoniae* strains, SB3341 (ST66-KL2) and NTUH_K2044 (SB3928; ST23-KL1), representing K1 and K2 capsule types, which are associated with severe invasive infections [6,7]. ST23 is the most frequent sublineage isolated in community-acquired liver abscesses, which is a prevalent infection in Asian countries [6,8,9].

We next selected seven capsule-deficient strains to be used for anti-K^d phage isolation. These included 6 *K. pneumoniae* and 1 *K. variicola* subsp. *variicola* (Kp3): Kp1 SB20*Δwza* ST15/O1v1 (04A025), Kp1 SB3928*Δwza* ST23/O1v2 (NTUH_K2044), Kp1 SB4021*ΔwcaJ* (SA1) and SB4454*ΔwcaJ* (CG43) ST86/O1v1; Kp1 SB4496*Δwza* ST380/O1v1 (BJ1-GA), Kp1 SB4975*Δwza* ST258/O2v2 (NJST258_2) and Kp3 SB579*Δwza* ST146-O3/O3a (342) [10]. Together, the O types of these strains represented 50.7% of non-redundant strains in the genomic database.

Supplementary text 2:

Small changes on phage genomes can lead to important host range differences

The anti-K phage cp48 and anti-K^d phage mtp6, turned out to have high sequence similarity (ANI: 99.8%) despite their different infection phenotypes (capsulated vs non-capsulated) and very different host-ranges (**Figure S13**). Sequence differences were detected in 3 different regions. Some SNPs were detected on two specific proteins and one 77 bp insertion was detected on an HNH endonuclease Gp2.8/Gp7.7 gene of phage cp48, disrupting the protein (proteins 43/44). HNH endonucleases have been shown to be associated with the terminases of a large number of diverse phages [11].

In order to explore the role of these differences on the phage protein structures, we performed a structural analysis of the altered proteins using Alphafold. The SNP detected on protein 2 of phage cp48 leads to an aspartic acid at position 261, while protein 62 of mtp6 contains an asparagine. This variant is not expected to lead to structural or functional differences on the protein as the amino-acids have similar properties. Comparative analysis with the NCBI protein database showed high similarity of the mtp6 CDS 62/cp48 CDS 2 to another phage particle-associated lyase. Despite no structural differences are observed, phage lyases are known to degrade specific membrane polysaccharides, which in this case may be the cause on the differential infection affinity observed towards the capsulated and non-capsulated strains. Further in-dept characterization of these proteins needs to be performed in the future.

In addition, a group of SNPs leads to a 7 amino-acids variation when comparing proteins 47 of cp48 and protein 18 of mtp6. This variation leads to important modifications on an exposed region of the proteins that could have functional implications: a highly positively charged loop in protein 47 of cp48 is replaced by a bulky hydrophobic loop in protein 18 of mtp6, with very different physico-chemical properties (Figure S13C). It is possible that these variations could play a role in the phenotypic differences between the two phages. However, we could not attribute a function to this protein (comparison by blastp with the NCBI database showed this protein to be closely related to other hypothetical proteins).

Resistance to the cocktail of cp48 and mtp6 phages was conferred by an interruption of the wcaJ gene by an IS5 (**Figure S12**), as previously observed when this strain and its capsule-deficient mutant were separately infected with cp48 and mtp6, respectively.

Supplementary figures:

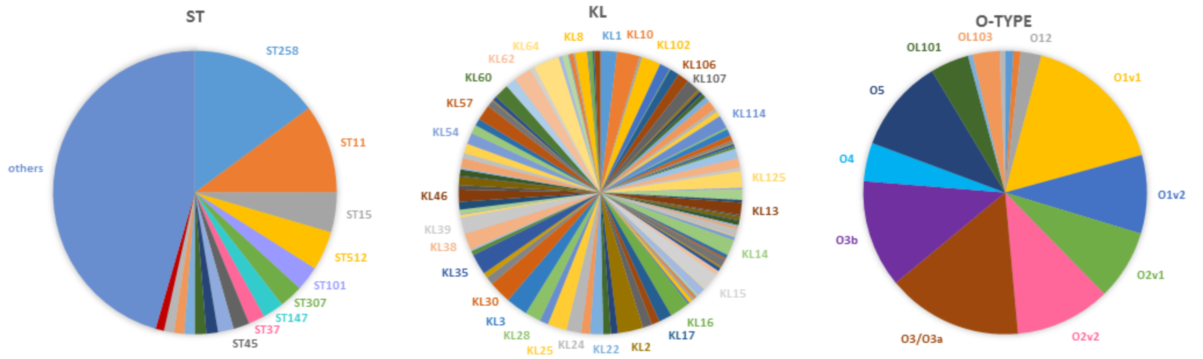

Figure S1. Frequency of *Klebsiella pneumoniae* genomes breakdown by ST, KL or OL characteristics. The ST distribution analysis was performed based on a dataset of 7388 genomes. To eliminate bias due to recent clonal expansions or outbreaks, KL- and O-antigen types frequencies were calculated using a non-redundant dataset of 1193 genomes, in which only one random strain per ST was included. Based on the MLST, the seven most prevalent allelic profiles present in the dataset (7,388 genomes) were ST258, ST11, ST15, ST512, ST101, ST307 and ST147. Analysis of the subset showed KL64, KL2, KL10, KL30, KL3, KL35, KL16 and KL102 to be the most prevalent capsular types, and the most frequent O-antigen types were O1v1, O3/O3a, O3b, O5 and O2v2.

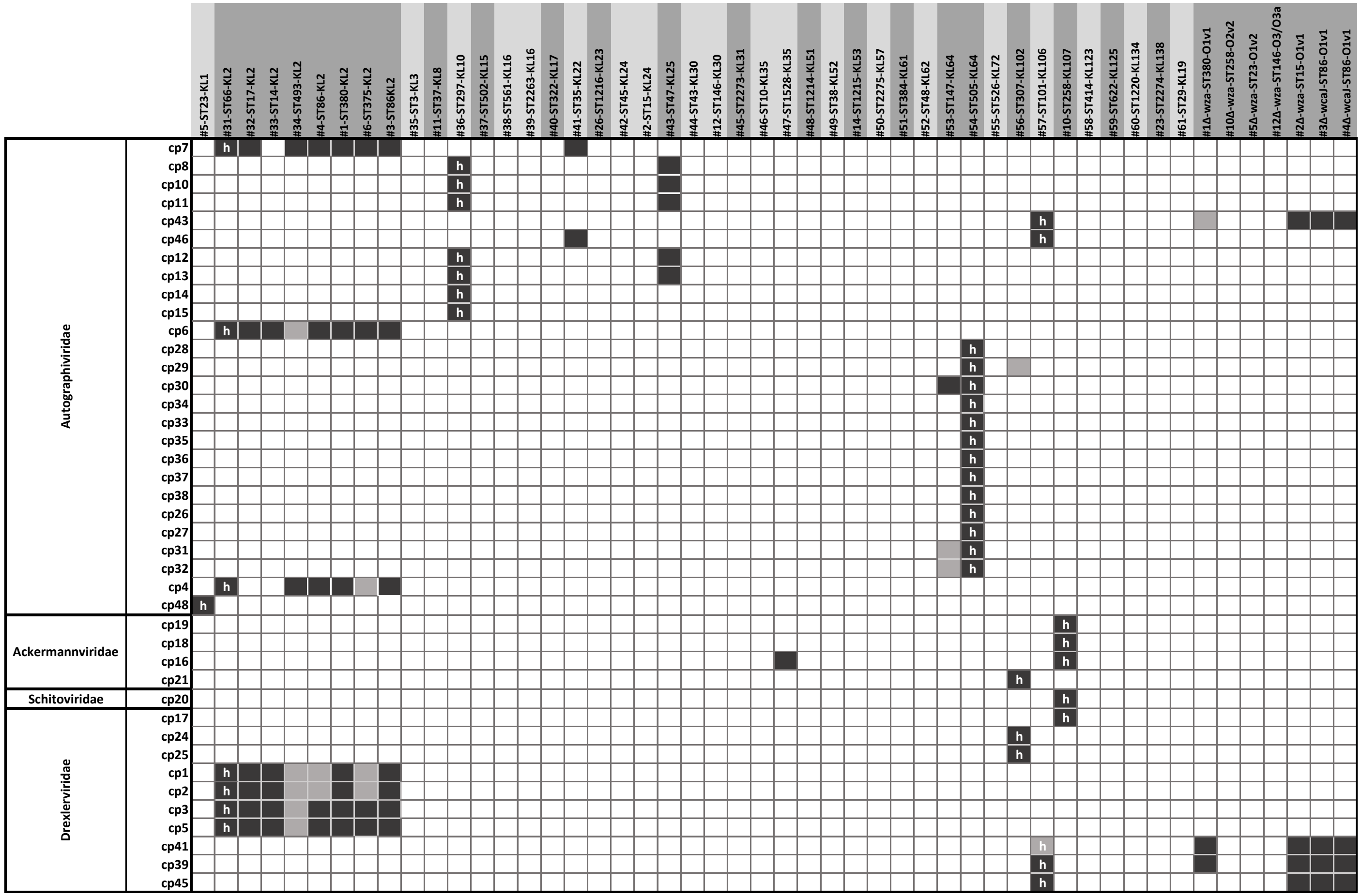

Figure S2. Host range of anti-K phages.

Anti-K phages were tested against wild-type strains and the 7 capsule-deficient (Δwza or ΔwcaJ) mutants. The dark-filled cells represent complete lysis, light-grey cells represent intermediate lysis and empty cells represent absence of lysis. “h” indicates the strain used as host for phage isolation.

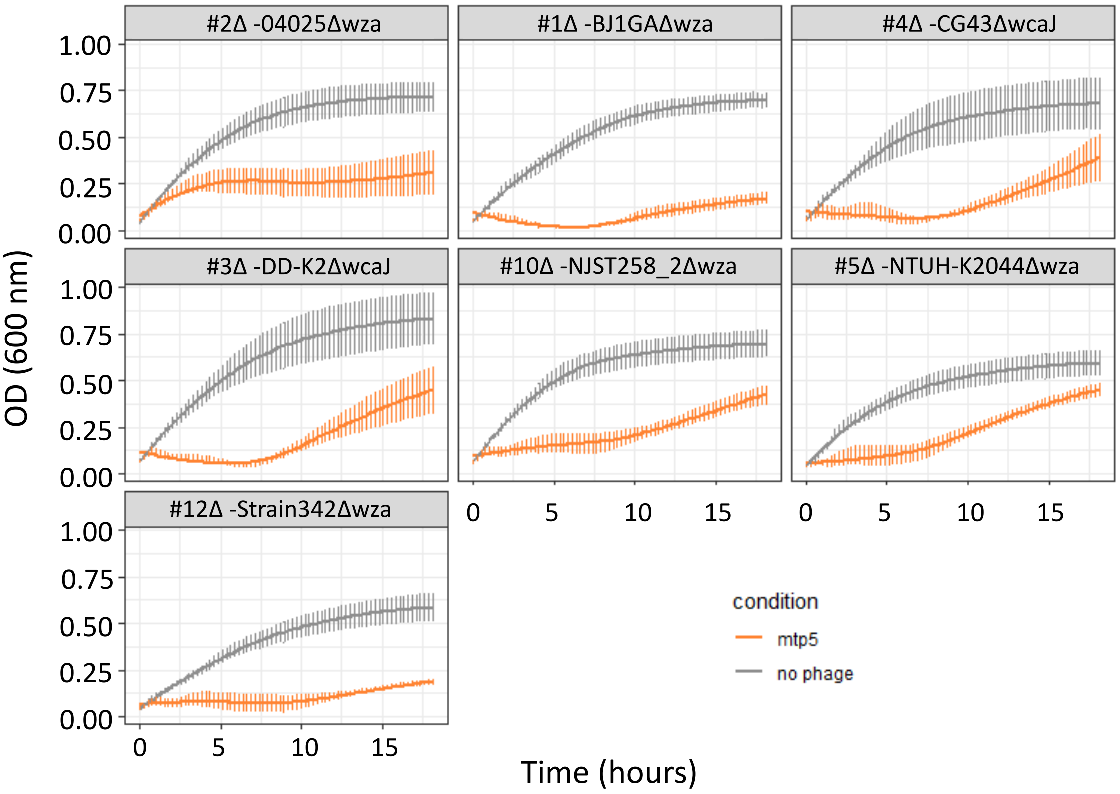

Figure S3. Lysis kinetics of phage mtp5 on the 7 capsule-deficient mutant (Δ*wza* or Δ*wcaJ*) strains. Growth curves of the seven capsule-deficient *K. pneumoniae* strains were calculated using three or more replicates for each condition; error bars represent standard error of the mean (SEM) measured by OD at 600 nm in liquid broth in the absence (grey) or presence (orange) of phage mtp5. At t = 0, the multiplicity of infection (MOI) was 10^2. X-axis: time in minutes.

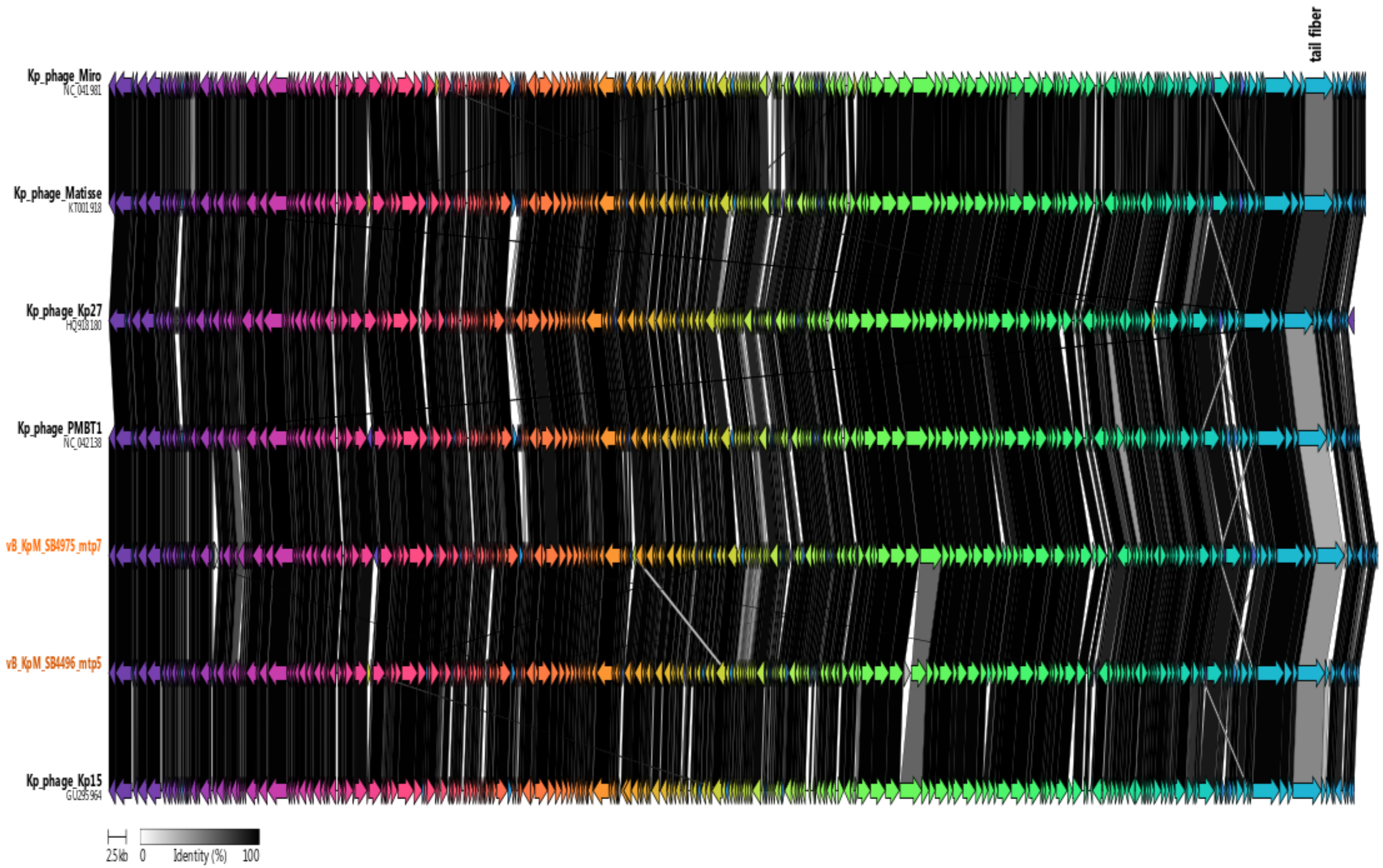

Figure S4. Anti-K^{d} phages mtp5 and mtp7 genomic similarities with closest anti-Klebsiella phages.

Phages Kp15, Kp27, Matisse, Miro and PMBT1 were identified based on BLASTN as the most similar to phages mtp5 and mtp7. Phages genomes were aligned using clinker. CDSs were arbitrarily colored. The white-to-dark grey blocks between CDSs represent their degree of aminoacid identity (see color scale at the bottom).

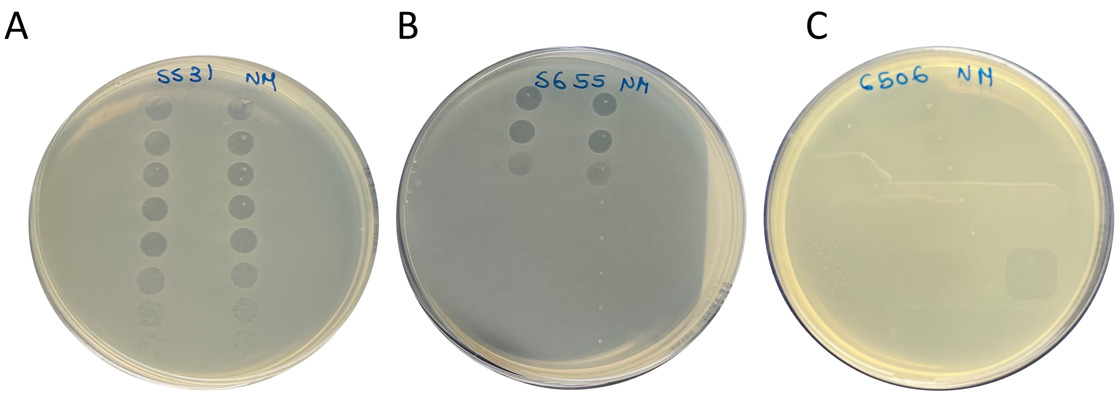

Figure S5. Example of titration of phage mtp5 on the generated spontaneous non-mucoid clones. When titration was performed on NM strains, we could observe, different infectivity phenotypes. Exponential liquid culture of NM clones was inoculated into a plate and mtp5 phage dilutions were spotted (4ul). A) titration on high-affinity strain, B) titration on strain with low affinity and C) no infection.

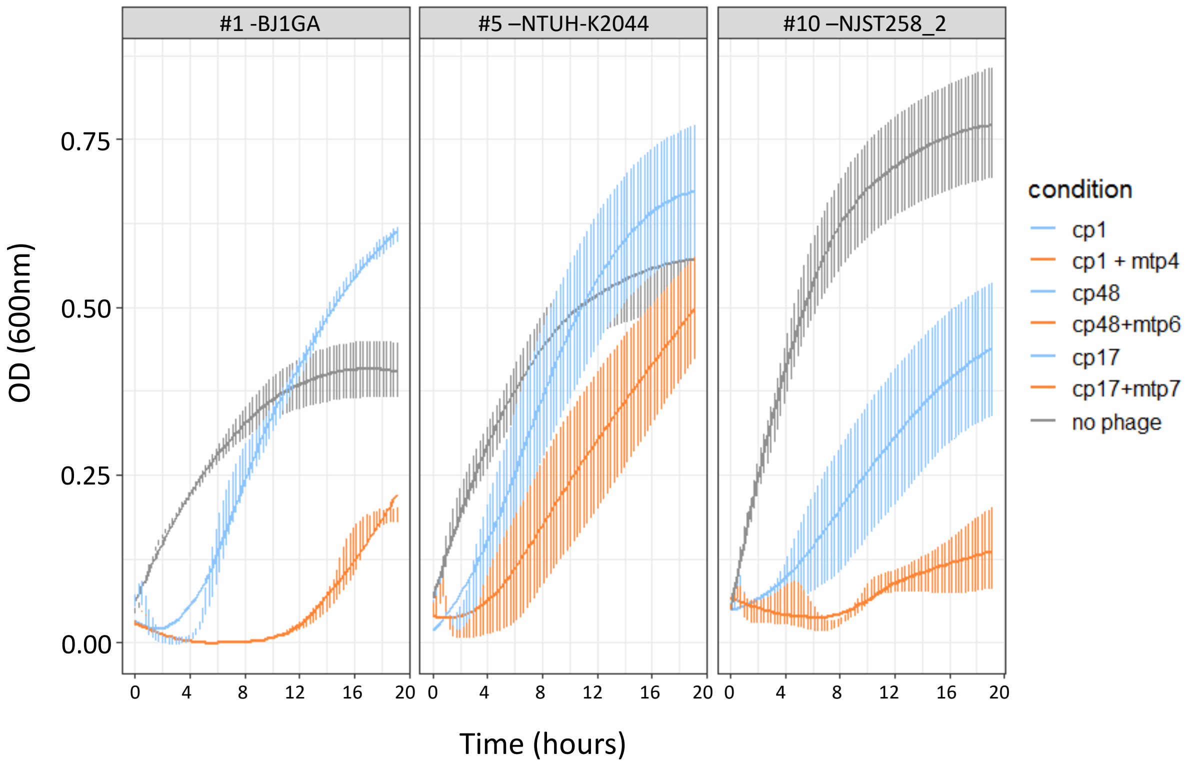

Figure S6. Cocktails of phages mtp4, mtp6 and mtp7 with anti-capsule phages

Growth curves for 3 different *K. pneumoniae* strains were obtained using three or more replicates for each condition; error bars represent standard error of the mean (SEM) measured via OD600 nm reading in liquid broth in the presence of three different phage cocktails. Cocktails containing anti-K phage and anti-K^d phages are in orange, whereas the use of anti-K phage alone is depicted in blue (grey: control without phage). Left, anti-K phage cp1 and anti-K^d phage mtp4 used against strain BJ1-GA; Center: anti-K phage cp48 and anti-K^d phage mtp6 used against strain NTUH-K2044; Right: anti-K phage cp17 and anti-K^d phage mtp7 tested against NJ-ST258-2.

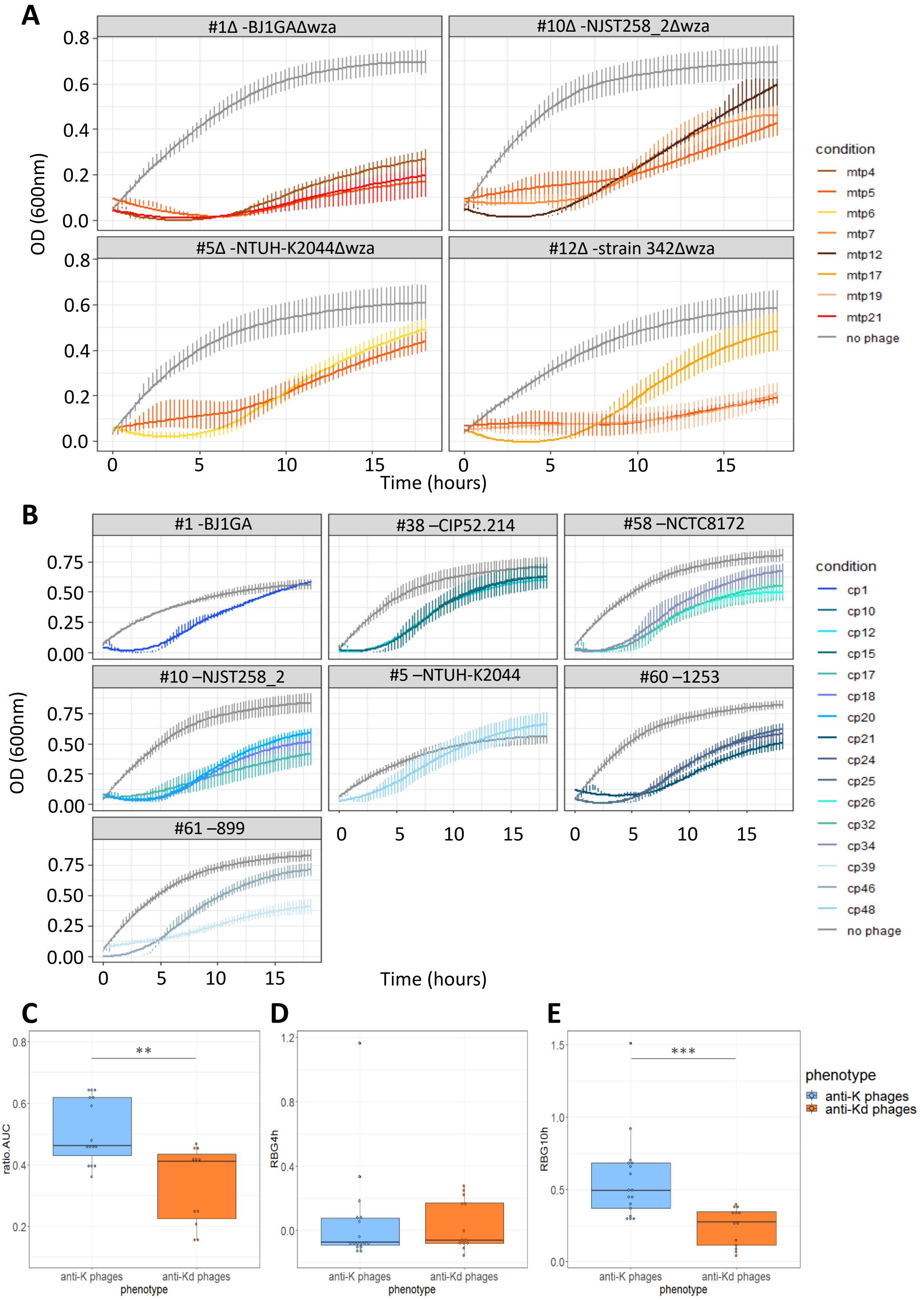
A
#1Δ -BJ1GAΔwza
#10Δ -NJST258_2Δwza
#5Δ -NTUH-K2044Δwza
#12Δ -strain 342Δwza
OD (600nm)
Time (hours)
condition
mtp4
mtp5
mtp6
mtp7
mtp12
mtp17
mtp19
mtp21
no phage
B
#1 -BJ1GA
#38 –CIP52.214
#58 –NCTC8172
#10 –NJST258_2
#5 –NTUH-K2044
#60 –1253
#61 –899
OD (600nm)
Time (hours)
condition
cp1
cp10
cp12
cp15
cp17
cp18
cp20
cp21
cp24
cp25
cp26
cp32
cp34
cp39
cp46
cp48
no phage
C
ratio.AUC
**
D
RBG4h
E
RBG10h
***
anti-K phages
anti-Kd phages
phenotype
anti-K phages
anti-Kd phages

Figure S7. Subpopulations of bacteria that are non-susceptible to phages emerge slowly when using anti-K^d phages against non-capsulated strains

A and B. Growth curves obtained using three Kp strains with or without capsule. Three or more replicates were used for each condition; error bars represent standard error of the mean (SEM) based on OD600 nm reading in liquid broth in the absence (grey) or presence of: **A.** anti-K^d phages (orange tones) and **B.** anti-K phages (blue tones). At t = 0, multiplicity of infection (MOI) was 10^2. In panels C, D and E, boxplots correspond to: **C.** Area under the curve (AUC); **D.** Relative bacterial growth (RBG) at 4h; and **E.** RBG at 10h, after the initiation of regrowth, on cultures targeted by anti-K phages vs anti-K^d phages (**P-value ≤ 0.01; ***P-value ≤ 0.001, Mann-Whitney test).

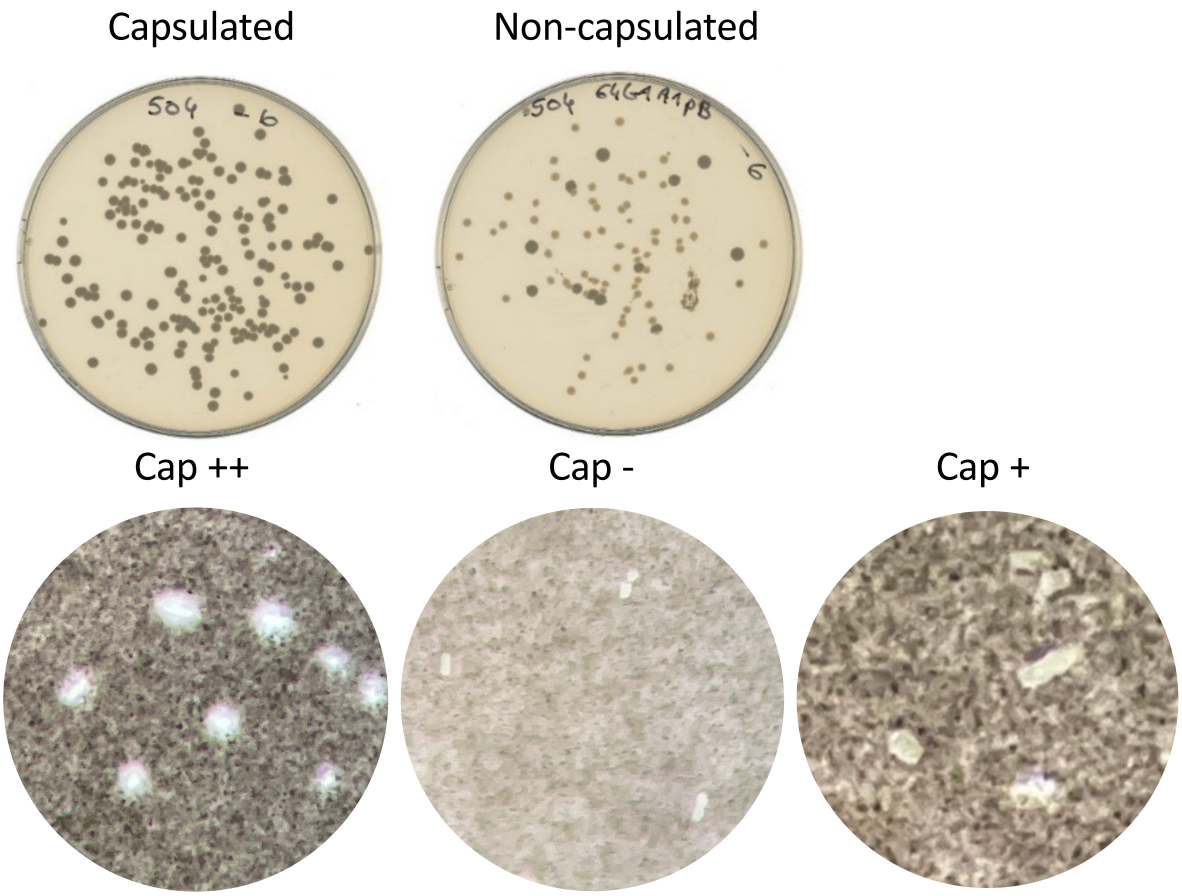

Figure S8. Resistance to anti-K phages leads to smaller (non-capsulated) colonies, contrasting with capsulated wt colonies.

Example of appearance of non-capsulated colonies after anti-K phage use against capsulated strains. Here, culture of K64 strain NCTC8172, comparing the initial and the derived population, which was non-susceptible to phage cp34. Capsule staining was also performed showing abundant presence of capsule on the "large" colonies, "medium" and non-capsule on "small" colonies (cap++ = hypercapsulated, cap - = non-capsulated, cap + = weak capsule).

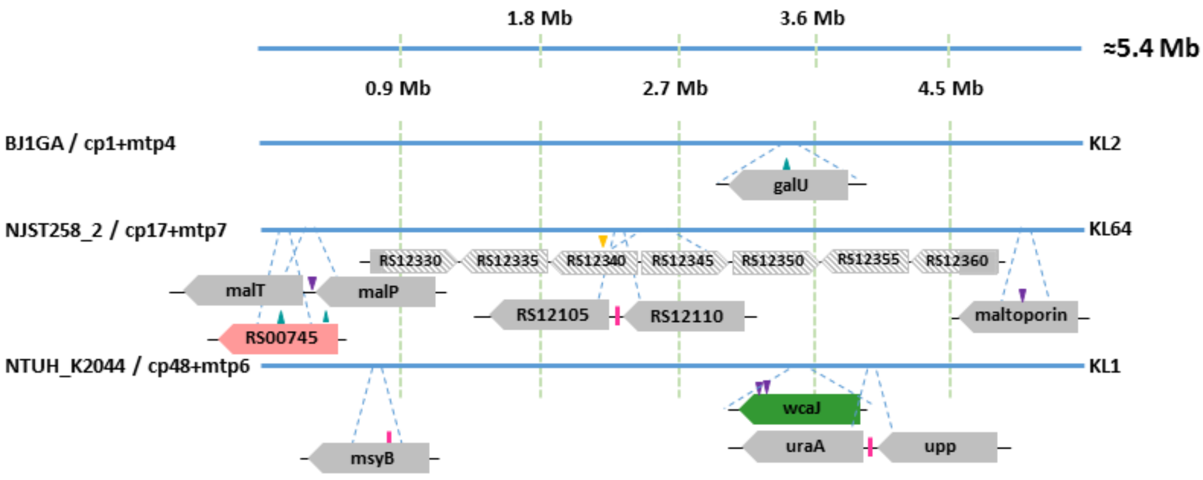

Figure S9. Mutations observed in populations exposed to phage cocktails that combine anti-K phages and anti-K^d phages mtp4, mtp6 and mtp7

Genes colors: green: capsule locus; pink: LPS locus; and grey: others.

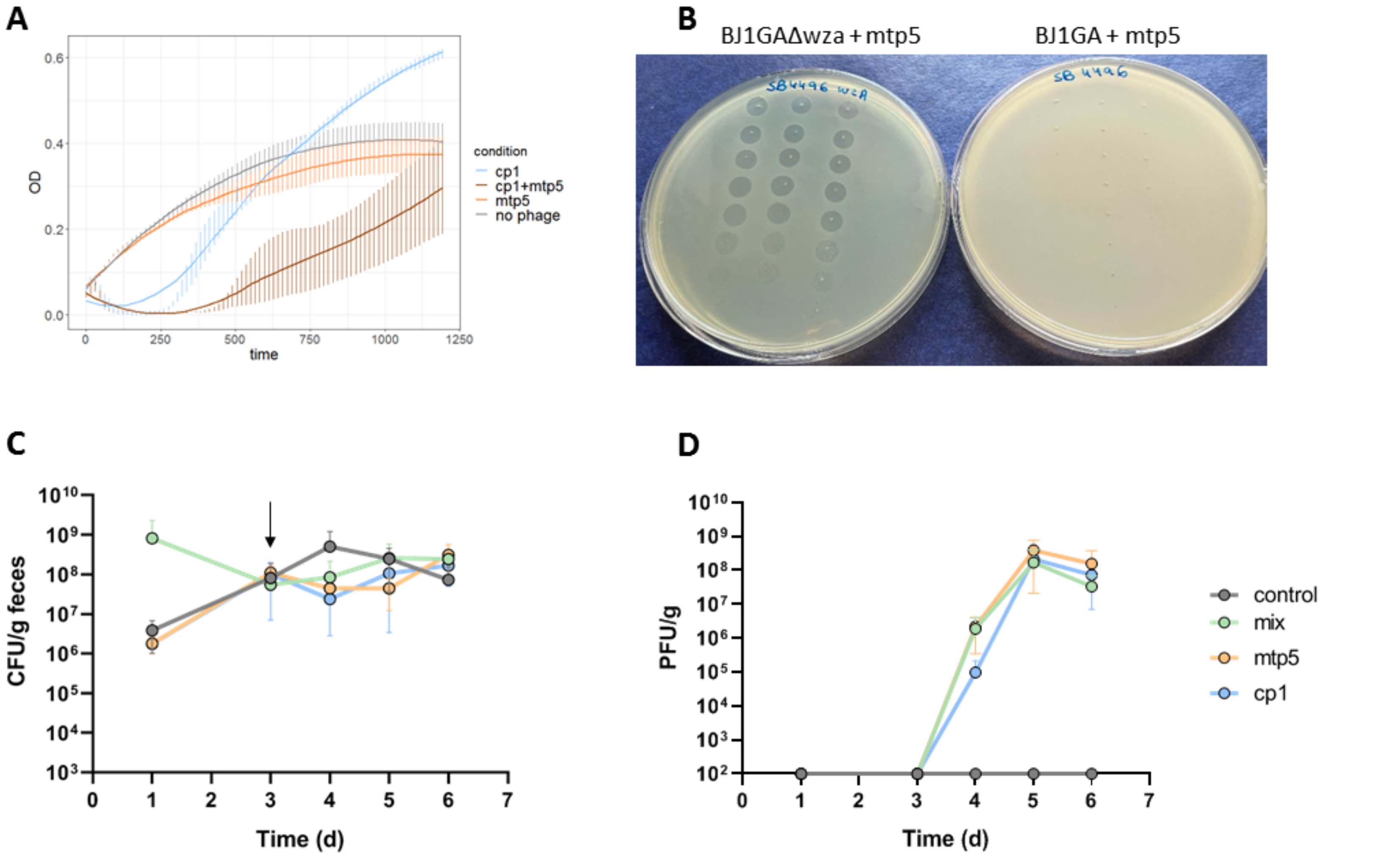

Figure S10. Phage mtp5 does not infect wild-type strain BJ1GA *in vitro* but infects it *in vivo*

A: Growth curves measured by OD600 nm reading in liquid broth for strain BJ1GA in the presence or absence of phages cp1 or mtp5. **B**: Bacterial lawns spotted with both phage types, showing that mtp5 does not infect strain SB4496 in *in vitro* solid media conditions. **C and D**. *K. pneumoniae*-colonized OMM12 mice

(n=13) received, only at day 3, either PBS (grey, n=4 mice), or the two phages cp1 and mtp5 together (mix; green, n=3; 6x10^7 pfu per dose made of the same amount of each phage), or the individual phages cp1 (blue, n=3) and mtp5 (orange, n=3) by oral gavage. **C.** Levels of *K. pneumoniae* BJ1GA strain in the feces (the arrow indicates the day the phage was given to the mice). **D.** Phage titers from the fecal samples reported in panel C.

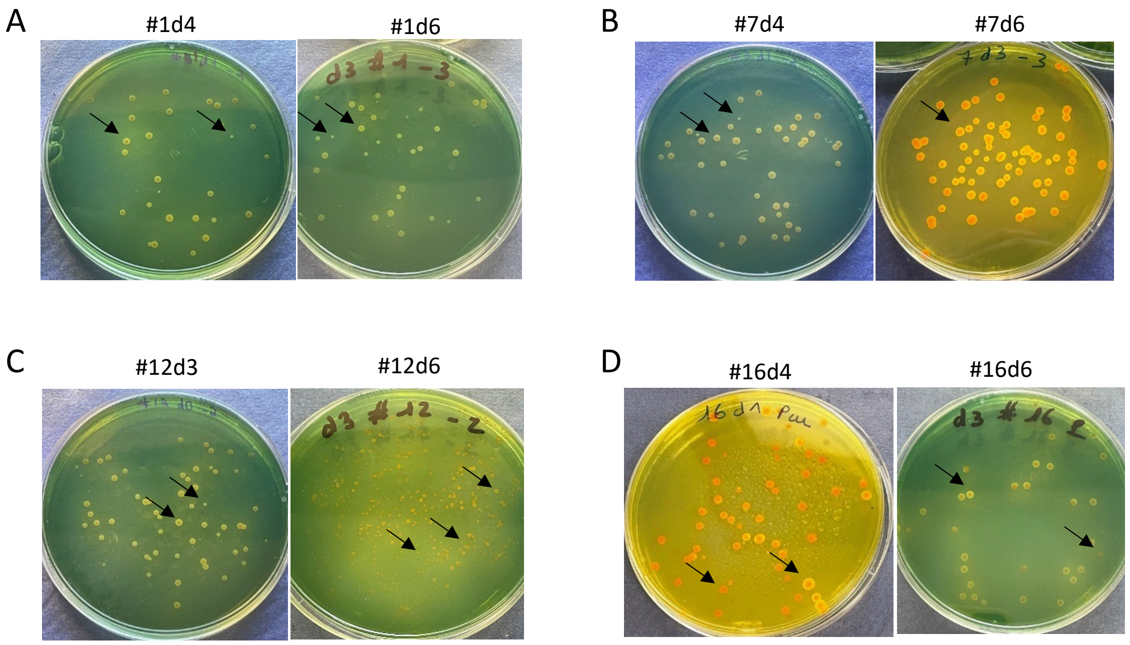

E

control mouse #1	cp1			mtp5		
phenotype	d4	d5	d6	d4	d5	d6
small	c2	c4	c6	c2	c4	c6
large	c1	c3	c5	c1	c3	c5

mtp5 mouse #7	cp1			mtp5		
phenotype	d4	d5	d6	d4	d5	d6
small	c8			c8		
large	c7	c9	c10	c7	c9	c10

cp1 mouse #12	cp1			mtp5		
phenotype	d4	d5	d6	d4	d5	d6
small			c18			c18
medium			c17			c17
medium	c11	c14	c16	c11	c14	c16
large	c12	c13	c15	c12	c13	c15

mix mouse #16	cp1			mtp5		
phenotype	d4	d5	d6	d4	d5	d6
small	c20	c23		c20	c23	c25
medium	c19	c22		c19	c22	c24
large		c21			c21	

susceptible	resistant	intermediate	not found

Figure S11. Isolation of different phenotypes from the feces of mice during phage treatment. **A-D**. Examples of the colony phenotypes isolated from the feces of mice after *K. pneumoniae* colonization and phage treatment. **A.** Two *K. pneumoniae* phenotypes (small and large colonies) observed on SCAI medium agar from mouse #1 of the control group. **B.** Example of the two phenotypes

isolated at day 4 (first day with phage) from feces of mouse #7 treated with phage mtp5 (the small phenotype was not observed the following days, at which only the large phenotype was observed. **C.** example of the three phenotypes isolated from feces of mouse #12 treated with phage cp1 (large, medium and small colony variants). **D.** Example of two of the three phenotypes isolated from feces of mouse #16 treated with cocktail of phages cp1+mtp5. **E.** All colonies (each being represented with a number preceded by the letter 'c', corresponding to those in table S10) were tested for resistance against the two original phages, showing different susceptibility patterns. Capsule staining presence/absence results can be found in table S9.

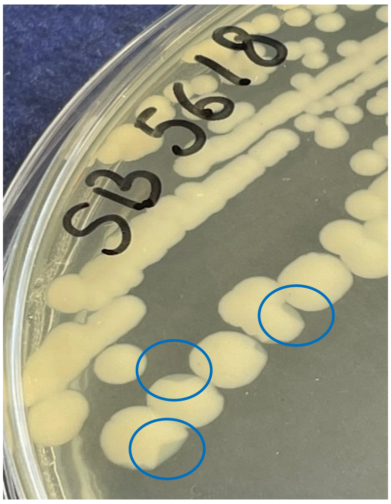

Figure S12. Example of non-mucoid sectors.

Capsulated (wild-type) *K. pneumoniae* strains were streaked on TSA (Tryptic Soy Agar) and incubated for 24 h at 37°C. The colonies were then searched for non-mucoid sectors; three examples are depicted on the plate. When none was detected, the plates were further kept at 25°C until non-mucoid sectors could be observed [12].

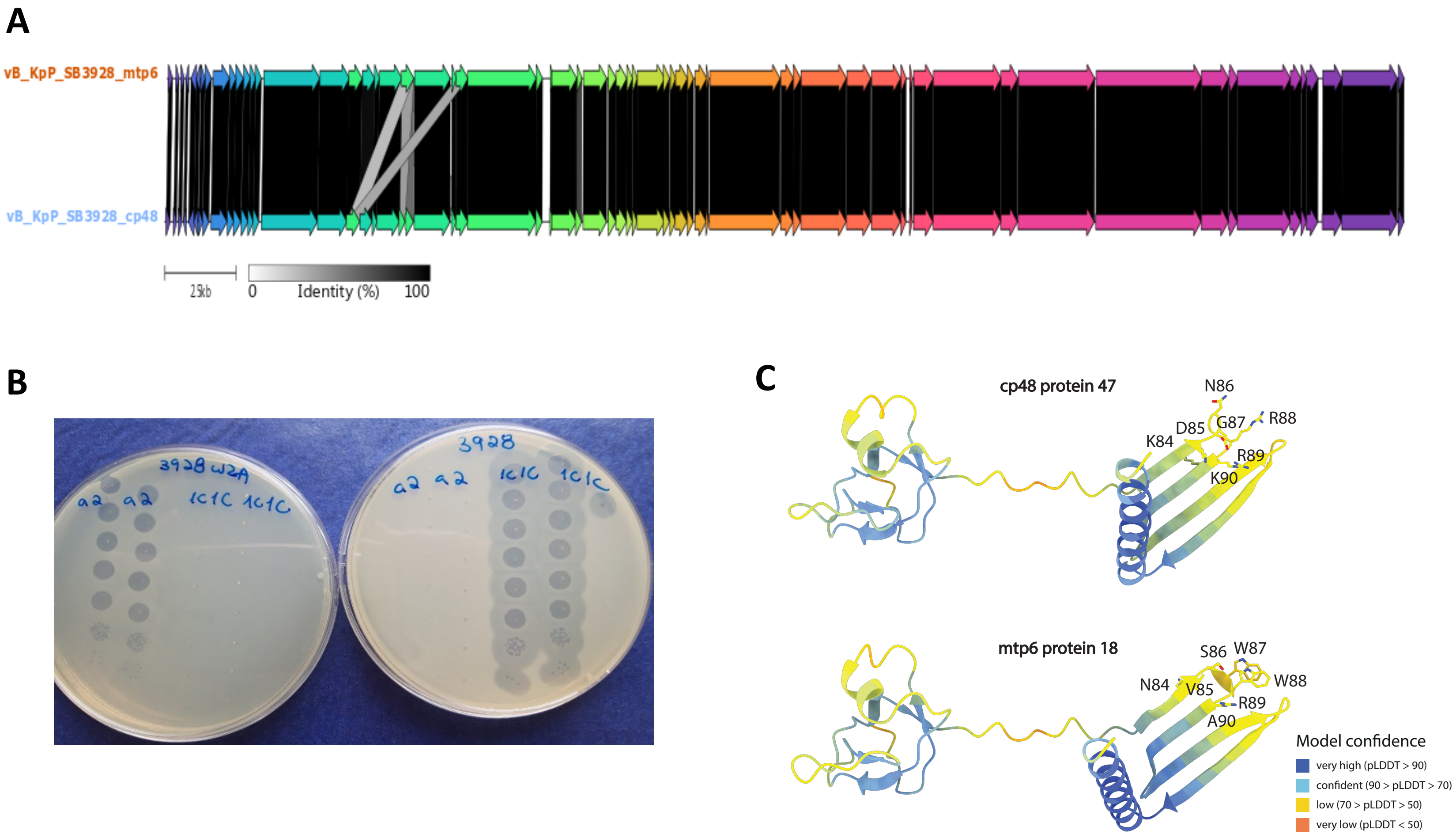
A
vB_KpP_SB3928_mtp6
vB_KpP_SB3928_cp48
2.5kb
0
Identity (%)
100
B
3928 W2A
a2 a2 1C1C 1C1C
3928
a2 a2 1C1C 1C1C
C
cp48 protein 47
N86
D85
G87
R88
K84
R89
K90
mtp6 protein 18
S86
W87
N84
V85
W88
R89
A90
Model confidence
very high (pLDDT > 90)
confident (90 > pLDDT > 70)
low (70 > pLDDT > 50)
very low (pLDDT < 50)

Figure S13. Comparison of phages mtp6 and cp48 for their genomic and proteic structures and infection phenotype.

A. Comparison of the genomic structure of phages mtp6 and cp48. The only differences are observed in two hypothetical proteins and in one HNH endonuclease (see supplementary text 2) **B**. Differences in infection phenotypes of phages mtp6 and cp48 using strain NTUH-K2044 (SB3928): mtp6 infects the non-capsulated NTUH-2044-wza mutant, and cp48 infects the wt strain. **C.** Alphafold predicted structural models of protein 47 of phage cp48 and protein 18 of phage mtp6, indicating the seven amino-acids that differ between the two proteins. These variants have very different physico-chemical propertied and are located in an exposed loop of the proteins, suggesting functional differences. Protein 47 of cp48 presents a highly positively charged loop (K84, R88, R89 and K90), while protein 18 of mtp6 has a bulky hydrophobic loop (W87 and W88). Predicted structural models are colored according to the Alphafold confidence estimate per-residue (pLDDT).

Supplementary references

1. Deleo, F. R. *et al.* Molecular dissection of the evolution of carbapenem-resistant multilocus sequence type 258 Klebsiella pneumoniae. *Proc. Natl. Acad. Sci. U.S.A.* **111**, 4988–4993 (2014).
2. Rodrigues, C., Desai, S., Passet, V., Gajjar, D. & Brisse, S. Genomic evolution of the globally disseminated multidrug-resistant Klebsiella pneumoniae clonal group 147. *Microb Genom* **8**, (2022).
3. Dong, N. *et al.* Genome analysis of clinical multilocus sequence Type 11 Klebsiella pneumoniae from China. *Microb Genom* **4**, (2018).
4. Huynh, B.-T. *et al.* Klebsiella pneumoniae carriage in low-income countries: antimicrobial resistance, genomic diversity and risk factors. *Gut microbes* 1–13 (2020) doi:10.1080/19490976.2020.1748257.
5. Wyres, K. L. *et al.* Emergence and rapid global dissemination of CTX-M-15-associated Klebsiella pneumoniae strain ST307. *J. Antimicrob. Chemother.* **74**, 577–581 (2019).
6. Wu, K.-M. *et al.* Genome sequencing and comparative analysis of Klebsiella pneumoniae NTUH-K2044, a strain causing liver abscess and meningitis. *J Bacteriol* **191**, 4492–4501 (2009).
7. Lery, L. M. S. *et al.* Comparative analysis of Klebsiella pneumoniae genomes identifies a phospholipase D family protein as a novel virulence factor. *BMC Biol* **12**, 41 (2014).
8. Turton, J. F. *et al.* Genetically similar isolates of Klebsiella pneumoniae serotype K1 causing liver abscesses in three continents. *Journal of medical microbiology* **56**, 593–7 (2007).
9. Merlet, A. *et al.* Primary liver abscess due to CC23-K1 virulent clone of Klebsiella pneumoniae in France. *Clinical microbiology and infection : the official publication of the European Society of Clinical Microbiology and Infectious Diseases* **18**, E338-9 (2012).
10. de Sousa, J. A. M., Buffet, A., Haudiquet, M., Rocha, E. P. C. & Rendueles, O. Modular prophage interactions driven by capsule serotype select for capsule loss under phage predation. *ISME J* **14**, 2980–2996 (2020).

11. Kala, S. *et al.* HNH proteins are a widespread component of phage DNA packaging machines. *Proc Natl Acad Sci U S A* **111**, 6022–6027 (2014).

12. Chiarelli, A. *et al.* Diversity of mucoid to non-mucoid switch among carbapenemase-producing Klebsiella pneumoniae. *BMC Microbiol* **20**, 325 (2020).
